# Large language models are able to downplay their cognitive abilities to fit the persona they simulate

**DOI:** 10.1371/journal.pone.0298522

**Published:** 2024-03-13

**Authors:** Jiří Milička, Anna Marklová, Klára VanSlambrouck, Eva Pospíšilová, Jana Šimsová, Samuel Harvan, Ondřej Drobil

**Affiliations:** 1 Institute of the Czech National Corpus, Faculty of Arts, Charles University, Prague, Czech Republic; 2 Department of Slavic and Hungarian Studies, Faculty of Language, Literature and Humanities, Humboldt University of Berlin, Berlin, Germany; 3 Faculty of Arts, Charles University, Prague, Czech Republic; University of Kurdistan Hewler, IRAQ

## Abstract

This study explores the capabilities of large language models to replicate the behavior of individuals with underdeveloped cognitive and language skills. Specifically, we investigate whether these models can simulate child-like language and cognitive development while solving false-belief tasks, namely, change-of-location and unexpected-content tasks. GPT-3.5-turbo and GPT-4 models by OpenAI were prompted to simulate children (N = 1296) aged one to six years. This simulation was instantiated through three types of prompts: plain zero-shot, chain-of-thoughts, and primed-by-corpus. We evaluated the correctness of responses to assess the models’ capacity to mimic the cognitive skills of the simulated children. Both models displayed a pattern of increasing correctness in their responses and rising language complexity. That is in correspondence with a gradual enhancement in linguistic and cognitive abilities during child development, which is described in the vast body of research literature on child development. GPT-4 generally exhibited a closer alignment with the developmental curve observed in ‘real’ children. However, it displayed hyper-accuracy under certain conditions, notably in the primed-by-corpus prompt type. Task type, prompt type, and the choice of language model influenced developmental patterns, while temperature and the gender of the simulated parent and child did not consistently impact results. We conducted analyses of linguistic complexity, examining utterance length and Kolmogorov complexity. These analyses revealed a gradual increase in linguistic complexity corresponding to the age of the simulated children, regardless of other variables. These findings show that the language models are capable of downplaying their abilities to achieve a faithful simulation of prompted personas.

## Introduction

As the language models are scaled up, new unexpected capabilities emerge. That means that the users (and sometimes even their developers) cannot predict in advance the full scope of their abilities based solely on their training objectives, as pointed out by [1]. Consequently, it becomes necessary to determine their capabilities post-hoc. Such studies are currently being conducted and published extensively in traditional scientific journals, preprints on arXiv, and blog posts.

Occasionally, a publication might assert that large language models (LLMs) are weak at some capability, only for someone else to use more effective prompting and demonstrate that the models are quite fluent at that task [2].

This inconsistency is not surprising, as currently, we are unable to probe the latent space of language models directly. Instead, we are limited to study agents based on these language models. The language model itself has no agency (in the traditional cybernetic sense, as used in [3]). We instantiate an agent by setting the initial conditions (*prompting*) and employ the model to predict continuation based on these initial conditions. If the initial conditions specify agents present in the model’s training dataset, the continuation serves as a simulation of those agents. Such agents are usually called ‘personas,’ although they are not only persons but can be whatever real or fictional agent the user needs: a *helpful AI assistant*, a thermostat, a hobbit, an ecosystem… What we typically refer to as the capabilities of a language model are, in essence, the capabilities of the persona simulated via the model. Naturally, these capabilities hinge on the model’s characteristics: whether its architecture is sufficiently versatile, if the training data encapsulates ample information on how the persona should behave, and whether the model can extrapolate a persona based on a combination of training data and prompt. However, the studied capabilities depend inherently on the attributes of the simulated persona.

**Fig 1:**
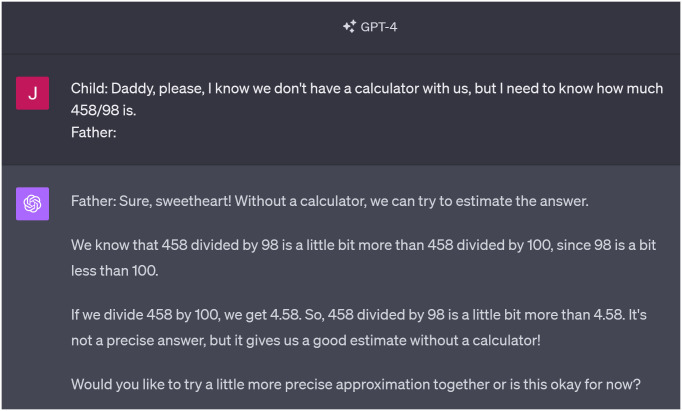
GPT-4 simulating a father who explicitly does not have a calculator at his disposal, default system prompt, 2023/10/9. Available at https://chat.openai.com/share/9c9e2954-b95f-4c79-9b2a-253218c6fa96.

For instance, if we interact with the default persona simulated by ChatGPT-4 and pose a simple arithmetic question, it answers correctly and precisely. However, if we craft a prompt to create a persona that explicitly lacks access to a calculator, it will behave as if it cannot arrive at an accurate result. This mirrors a real human’s behavior without a calculator (see Fig 1).

These observations and terminology are based on the *simulation theory*, initially introduced by a pseudonymous blog post by Janus [4] and later elaborated upon in scholarly literature [5]. The theory explicitly states that the agency resides not in the model itself but in the simulacrum instantiated by the prompt. As [5] references [4], “To better reflect this distributional property, we can think of an LLM as a non-deterministic simulator capable of role-playing an infinity of characters, or, to put it another way, capable of stochastically generating an infinity of simulacra”.

Some language model interfaces permit users to construct personas purely based on their own prompting (e.g., Davinci-002 model by OpenAI, accessible via API), while others provide a default persona (e.g., the *helpful assistant* in ChatGPT). In the case of ChatGPT, the final persona is collaboratively shaped through additional reinforcement learning from human feedback (RLHF), an unchangeable system prompt, and finally, the interactions initiated by the user.

When users or researchers evaluate LLMs, it is crucial to recognize the role of the specific persona being simulated at that moment. Claiming that a language model lacks a certain capability is an inappropriate act of anthropomorphization. It is preferable to assert that the personas, simulated with the assistance of a specific language model, lack a certain capability (even though they should possess it in the real world). Anthropomorphizing human-like simulacra can be a useful shorthand, assisting us in predicting their behavior, in contrast to anthropomorphizing language models themselves [2].

That leads us to the primary focus of this article: To what extent can we effectively employ LLMs to simulate personas characterized by limited cognitive capacities? Certainly, the cognitive abilities of the personas cannot surpass the limitations inherent to the architecture and training data of the model. For instance, models like Ada-002 (by OpenAI) have a ceiling when performing basic arithmetic operations beyond a certain threshold, even if they are quite adept at mimicking mathematics educators. While this upper limit has been extensively investigated and discussed in numerous articles [6], our current interest lies in the opposite question: Are we able to credibly simulate personas that have cognitive abilities considerably below this limit? Can we use LLMs to simulate inabilities that are subtler than, for instance, a persona lacking a calculator as in Fig 1?

The human brain is capable of this simulation, meaning that people can downplay their cognitive abilities. This study tests the hypothesis that LLMs possess similar adaptive capabilities.

It is necessary to examine personas that are, by definition, differentiated by their cognitive abilities and whose behavior is represented in the training data of existing LLMs. We selected children of various ages as ideal personas since a child’s cognitive level depends on their age, and these differences are well-documented in the vast body of cognitive developmental literature. Observing simulated children, we will monitor two competencies:

**Linguistic Ability**: We are interested in how the language model can adapt the complexity of linguistic expression to match the presumed abilities of the simulated speaker.
**Mental State Understanding**: We test the ability of the simulated personas to simulate the mental states of other entities.

We chose the Theory of Mind (ToM) framework as an ideal candidate for the exploration of mental state understanding since it showcases pronounced age-related differences in performance, providing us with a gradient to study. Also, the performance in so-called false-belief tasks (a common way of testing ToM) is well-studied and documented in the cognitive developmental literature (see, e.g., [7] for an overview).

For both competencies, linguistic and cognitive, we anticipate a progressive increase of capabilities correlating with the increasing age of the simulated child personas. This progression would ideally mimic real-world linguistic and cognitive developmental patterns observed in children. If LLMs can effectively simulate this gradient of development in their responses, it would validate their capability to adapt to different cognitive levels based on the prompts provided.

### State of the art

Recent works on LLMs’ ability to simulate human reasoning and cognitive skills have brought mixed findings. [8] identified an ability of GPT-3.5 to manifest human-like intuitive behavior in various cognitive tests. [9] found LLMs failing in abstract reasoning in a similar manner as humans. LLMs’ ability to understand and simulate ToM has been investigated. ToM is the ability to understand and reason about the mental states and intents of others. It enables people to predict behavior and understand the reasoning of others [10]. [10] tested GPT-3 in the variation of a classical false-belief task [11, 12] and discovered that compared to 90–100% accuracy of human subjects, GPT-3 models peaked at 60% accuracy. In a social commonsense and emotional intelligence test, the model achieved 55% accuracy, compared to more than 85% accuracy of human subjects. [13] compared responses of several LLMs to false-belief tasks (‘unexpected transfer’ and ‘unexpected content’) and discovered a high improvement in the accuracy of solving these tasks in ChatGPT-4. In their experiment, the accuracy of Davinci-001 and Davinci-002 was 10%; Davinci-003 and GPT-3.5-turbo achieved 35% of accuracy; and GPT-4 solved successfully 90% of the tasks. The authors theorized that ToM has spontaneously emerged as a byproduct of rapidly improving the language skills of LLMs.

The studies mentioned above simply asked the LLMs the questions to gain the responses; they did not prompt the models to simulate some specific persona. [14] introduce a so-called *Turing experiments*. In these experiments, the LLM is prompted to simulate personas, which then serve as human participants. Therefore, the question that these experiments can answer is not if an LLM is able to answer tasks as humans but if it can faithfully simulate aspects of human behavior. The authors claim that these simulations should be zero-shot. They applied this design to *Ultimatum Game* (a share of money is offered to a participant, who can either accept or reject it based on the appropriateness of the split), *Garden Path Sentences* (participant has to decide if a sentence with confusing parsing is grammatical or not), *Milgram Shock Experiment* (participants are instructed to punish a learner with an electric shock if the learner does not perform well), and *Wisdom of Crowds* (participants have to estimate a numerical value of a general-knowledge question) tasks, and they yielded results comparable to human subjects in the three first test, including gender-sensitive chivalry effect in the Ultimatum game. Additionally, they found surprising hyper-accuracy distortion for the Ultimatum game in the recent GPT models. They explain this distortion as a product of the alignment procedures, namely Reinforcement learning from human feedback (RLHF), which are not present in older models.

Using LLMs as representations of humans with various demographic properties has been suggested by other researchers (e.g., [15–19]).

Following this research approach, our inquiry extends beyond evaluating the capacity of Large Language Models (LLMs) to mimic human behavior. It also encompasses the examination of their ability to simulate the limitations in cognitive abilities. While it has been discovered that some LLMs exhibit ToM and can answer false belief questions similarly to humans, our interest now lies in their ability to conceal their capabilities when asked. To investigate this, we have prompted LLMs to simulate individuals with incomplete cognitive development: children.

It is well-documented that children acquire aspects of ToM from their surroundings, as evidenced by established assessments such as false-belief tasks. Comprehensive meta-analyses of ToM studies with children are available [7, 20–22], and for a broader perspective on these meta-analyses, refer to [23]. Although there is a prevailing trend indicating an enhancement in children’s ability to correctly solve false-belief tasks as they age, it is essential to recognize the presence of individual differences. These variations are attributed to various factors, including having siblings, frequently engaging in social-pretend play, or being bilingual [23]. False-belief tasks have proven to be effective assessments of ToM abilities since they replicate everyday scenarios easily comprehensible to children. The results of the meta-analysis have revealed that the specific type of false-belief task employed does not significantly impact children’s responses. Among the commonly used false-belief tasks are the change-of-location and unexpected-content tasks [7, refer to Section ‘Prompt type’ for further information]. The progress of correct solving of those tasks is rapid between 2.5 and 5.5 years, after which it slows down. By 4.5 years of age, most children assess and reason in those situations correctly [23].

In addition to cognitive development, there is a concurrent improvement in language skills. These advancements are well-defined by Brown’s stages of syntactic and morphological development, which establish the expected expressive language abilities in children aged approximately 1 to 4 years [24]. One crucial metric for measuring language development is the Mean Length of Utterance (MLU), which gradually increases within this age range. Children typically produce utterances ranging from 1.0 to 2.0 morphemes at one year of age, which progressively extend to 3.75–4.5 morphemes by the age of 4. While MLU has faced some critique (e.g., [25–27]), it has remained a valuable indicator of language development (e.g., [28–30]). The reflection of linguistic development extends beyond MLU; it encompasses overall complexity. This complexity involves constructing more elaborate sentences and conveying meanings with greater precision [31]. The developmental stages summarized by [32] describe that first language production typically occurs around six months of age with syllable repetition, followed by the emergence of single words around one year of age. By two years of age, children combine two words, leading to the production of short sentences at three years, complex sentences at four years, and, eventually, the ability to narrate a brief story after five years of age.

### Hypothesis

Our hypothesis arises from the endeavor to determine whether current language models inherently embed maximum cognitive abilities into simulated personas regardless of the context, or if they instead aim to faithfully simulate personas, including their cognitive imperfections. The motivation behind this is to investigate the possibility that, with the rapid development of models, we might reach a stage where we fail to leverage their full potential, since models’ capabilities are deliberately constrained to simulate human or human-like personas, as dictated by the prompt.

The central thesis of this paper can be summarized as: *It is possible to set initial conditions for large language models such that the resulting simulated personas differ from each other in cognitive and linguistic abilities, and these differences are in accordance with the differences between the entities in real life*.

Our primary hypothesis posits that LLMs are capable of simulating a deficiency in cognitive and linguistic capacities. In other words, even though they possess certain capabilities, they can simulate personas lacking those abilities. By simulating children, i.e., personas with underdeveloped cognitive and linguistic skills, our aim is to investigate whether LLMs can accurately mirror the skills exhibited by a typical human child of a specific age. To test this hypothesis, we will focus on two key variables: language complexity, serving as an indicator of language skills, and the accuracy of responses to false belief tasks, which provides insights into the understanding of ToM. Performance in these two domains gradually improves during a child’s development, enhancing cognitive task capabilities and the complexity of language production. We predict that LLMs will replicate this developmental trajectory within the simulated personas.

## Methodology

We operationalized testing this hypothesis in the following manner: We prompted LLMs, initiating a conversation designed to simulate a child’s discussion with an adult. The primary independent variable under consideration was the child’s age. Outcomes were assessed regarding linguistic complexity and cognitive performance as the dependent variables.

Since the results may be influenced by numerous other independent variables, some of them were systematically manipulated to explore the latent space of the models in question. This approach resulted in a total of 1296 independent trials. In each trial, LLMs had the opportunity to generate all intermediate responses, not just a final answer, thereby simulating a genuine dialogue.

Given that the contemporary Western LLMs are predominantly trained on English data, the entire experiment was conducted in English. That also allowed us to utilize the extensive corpora of the child’s language in English, which is a part of the CHILDES data bank [33].

### Independent variables

#### Age

We concentrated on children and personas aged 1–6 years, with a granularity of one year. This age range was chosen due to the critical developments in language skills [31] and understanding of ToM [7, 20–23] that occur during this period.

We used data from the following corpora: Bates [34], BernsteinRatner [35], Brown [24], Demetras—Trevor [36], Gelman [37], Gleason [38], Higginson [39], HSLLD [40], McCune [41], and Morisset [42]. When extracting data, we ensured that children in the 2, 3, 4, and 5 age groups were no more than two months older than the target age. For 6-year-olds, we selected transcripts from children aged 6 years to 6 years 6 months. Regarding 1-year-olds, we opted for children at the age of 1 year 6 months, as transcripts from younger children were scarce and lacked replicas of child speech.

#### Prompt type

We presented LLMs with three prompt types:

(1) Plain zero-shot prompting: The conversation starts with the line “Here is a transcript of a conversation between a X-year-old child and her/his mother/father”. The age and the gender of the child and the gender of the parent were manipulated (more below). The task scenario followed immediately after this introduction.

(2) Chain-of-thought prompting: In this case, the conversation starts with the following prompt: “You are an expert in the field of children’s psychological development, possessing comprehensive knowledge in both the theoretical and practical aspects of their language and cognitive abilities as understood by science. Could you please share your insights about the theory of mind in children? What is it exactly, and how does it vary with the child’s age?” After an LLM generated the answer, the following prompt appeared:

Using this information, please continue a conversation between a child of X years and her/his mother/father in the following transcript: The gender of the child and the parent and the age of the child were manipulated. After that prompt, one of the task scenarios followed.

(3) Primed-by-corpus prompting: The conversation starts with an excerpt from CHILDES corpus (more in Laboratory protocol (S1 File). Each excerpt consisted of approximately 100 replicas, with a permissible variance of ±5 replicas. Notably, no explicit information regarding the child’s age was provided within these passages. Ten excerpts from CHILDES were selected for each age group, and they originated from English-speaking individuals who were monolingual. After the excerpt, the task scenarios followed.

Plain zero-shot prompting follows the procedure suggested by [14]. Chain-of-thought prompting was employed since previous studies using this methodology report improvement in the precision of results [43, 44]. We modified this methodology to align with our research objectives. Specifically, we diverged from the standard practice of requesting the generation of intermediate steps and instead focused on soliciting explicit recall of theories that would subsequently be applied to our tasks. In this case, the LLM, therefore, simulates an expert who simulates a child. Lastly, primed-by-corpus prompting was chosen to prompt the model with implicit rather than explicit information about the desired persona.

#### Task

We chose two most common false belief tasks in ToM research: (1) Change-of-location task: We follow a classical scenario suggested by [12]. The series of prompts is as follows:

**Parent:** Can you remember Maxi, your friend?
**Child:**
**Parent:** Here is a puppet. The puppet is like Maxi, isn’t he?
**Child:**
**Parent:** Maxi has a chocolate, here is his chocolate… And Maxi, here, puts the chocolate in the cupboard.
**Child:**
**Parent:** Now Maxi left! Maxi went to a playground.
**Child:**
**Parent:** And here comes his mommy to the cupboard! Here this puppet is his mommy. And she takes the chocolate!
**Child:**
**Parent:** And she gives the chocolate to a drawer. Here.
**Child:**
**Parent:** Now Maxi is back from the playground! And he wants the chocolate. Where will Maxi look?
**Child:**

While 3-year-olds often fail this task, from 4 years of age, children become mostly capable of recognizing that Maxi did not see the parent hiding the chocolate. Therefore, they answer correctly that he will look into the cupboard [20].

(2) Unexpected-content task: This task is a modification of a scenario suggested by [45]. It differs from the change-of-location task because the child experiences the false belief on their own, believing that there are candies in the candy box before the revelation that there are, in fact, pencils. The series of prompts are as follows:

**Parent:** I have something for you!
**Child:**
**Parent:** here! look at this candy box!
**Child:**
**Parent:** what do you think is inside?
**Child:**
**Parent:** but see! When I open it, there are pencils inside!
**Child:**
**Parent:** I will close the box now and I will show it to your twin sister, ok?
**Child:**
**Parent:** what will your sister think is inside the box?
**Child:**

Though lacking certainty, it is plausible to assume that these types of tasks were available in the training data of LLMs. These scenarios are widely recognized examples, which was a crucial aspect of the study, as the aim was to juxtapose the answers of personas with the performance of children. Regarding the potential impact of specific tasks on the results, the large meta-analyses did not discover a significant effect of the task type on the correctness of the answers [7, 20]. Consequently, we can anticipate comparable results across both tasks.

#### Gender of child and of parent

While our primary focus did not revolve around the influence of gender, research in language acquisition has revealed correlations between a child’s gender and their language skills. Specifically, it has been observed that girls often exhibit a slight advantage over boys in this regard (e.g., [46–48]), and boys are recognized as a group with greater variability [49]. Furthermore, various aspects of communication, including narration style, choice of speech acts, and negotiation methods, appear to differ according to gender [31]. Consequently, we aimed to ensure a balanced representation of gender among our personas. Since some studies showed differences between input from female and male caregivers, e.g., [50, 51], we balanced the gender of the parent as well.

The balancing of the gender was carried out exclusively in the plain zero-shot and chain-of-thoughts prompting, where we had the flexibility to manipulate the explicit information provided in the initial prompt. However, this was not a viable option in the primed-by-corpus prompting method for several reasons. Firstly, the available transcripts exhibited an asymmetrical representation of female caregivers over male caregivers, and the gender distribution among the children was also unbalanced. Secondly, the transcripts required manual selection, as some children had fewer than two replicas in the randomly chosen excerpts. Lastly, the primary purpose of this prompting method was to imply the child’s age through behavioral data, and most transcripts did not contain gender information. Implementing gender information into all transcripts would have necessitated altering the original behavioral data, a step we chose not to take. Additionally, pragmatically, we did not have access to sufficiently large corpora to ensure gender balance in this method.

#### Models

We employed GPT-3.5-turbo and GPT-4 because of their capabilities, popularity, common real-world usage, and easy access through the API. As of writing this article, GPT-4 is the most advanced publicly available language model [52].

Although these models are termed large language models, they are utilized more as cognitive co-processors or reasoning machines. In this context, language primarily serves as an interface, and other cognitive abilities are more important. Currently, other cognitive capabilities are being tested more extensively than the linguistic ones, c.f. [52].

Reinforcement Learning from Human Feedback (RLHF) enhances the cognitive abilities of LLMs in certain tasks that are in demand and makes the model more pleasant to use [53]. However, applying RLHF in GPT-4 has been identified as a potential reason for certain anomalies, particularly in the manifestation of hyper-accuracy [14]. The distinction between GPT-3.5-turbo and GPT-4 is not just in their size and architecture (of which GPT-4’s hasn’t been disclosed to general public), but also in the nature and degree of RLHF they underwent.

#### Temperature

The model determines the likelihood of each possible token being a continuation of the given text (note that in language modelling termonology, *token* means *character n-gram selected as a tokenization unit*, not a part of Peirce’s type-token distinction, in which case *likelihood of each possible type* would be more appropriate). Subsequently, an output token is randomly selected from this probability distribution. This selection mechanism is skewed, favoring more likely tokens to some extent. The degree to which this occurs can be adjusted based on API user requirements, using the *temperature* parameter. When the temperature is set to 0, the token with the highest probability is always chosen. As the temperature value increases, less likely tokens also have a chance to be selected. In the OpenAI API documentation, it is explicitly stated: “Higher values like 0.8 will make the output more random, while lower values like 0.2 will make it more focused and deterministic” [54].

For the purposes of our experiment, we selected three temperature values to explore how the model’s properties change depending on this crucial parameter. The first value is chosen as zero, representing deterministic selection of the most probable token. The second is 0.5, which lies in the middle of the suggested range for standard use. The third selected value is 0.9, exceeding the typical suggested range.

### Dependent variables

#### Language complexity

We utilized two distinct methods for assessing complexity: first, an approximation based on response length, and second, an approximation of the Kolmogorov complexity.

Response length was chosen as a metric because of its prevalent use in the literature on primary language acquisition (see above), maintaining continuity with established tradition. This measure is employed in four figures representing the principal findings in the Results section. Our operationalization of this measure is the count of letters in the text generated by the model as a child’s response. We acknowledge that psycholinguistic studies often employ various other operationalizations (e.g., morpheme count), but this simple metric suffices for our purposes.

The Kolmogorov complexity offers more precise measurement and is widely used in quantitative linguistics [55]. Kolmogorov complexity, also known as algorithmic complexity [56, 57], refers to the minimum amount of information required to compress a given string. As such, it cannot be calculated directly, but only approximated. We approximate the upper bounds of the Kolmogorov complexity using text compression via a combination of the LZ77 algorithm [58] and Huffman coding [59], amalgamated into RFC1951 [60]. We include only one figure in the main article based on the results of this measurement, but further results can be found in the Supporting Information (S3 File).

We only consider the simulated persona’s responses, excluding parentheses, remarks, and annotations.

#### Theory of mind

The answers to the two false-belief tasks were analyzed to assess the ToM understanding of the personas. The first reply following the question “Where will Maxi look?” (or What will your sister think is inside the box?, respectively) was classified into four categories as displayed in Table 1.

**Table 1:** Overview of the classification of the LLMs answers to the false-belief tasks.

	Change-of-location	Unexpected-content
	Where will Maxi look?	What will your sister think is inside the box?
nothing	eg., I don’t know!	not answering the question, eg., Surprise!; I want to find out!
mastered ToM	cupboard	candy/candies
failing ToM	drawer	pencils
something else	eg., in the kitchen	eg., toys, stickers, or listing options

The responses provided by the personas underwent manual coding by an experienced coder. To ensure the reliability of the coding process, 30% of the responses were independently coded by a second coder following the established rules outlined above. Intercoder reliability was assessed using Cohen’s kappa index. The calculated value of Cohen’s kappa index between the two coders was 0.88, indicating that the level of agreement between the coders was ‘almost perfect,’ according to the benchmarks for assessing agreement strength proposed by [61]. There was a 95% agreement between the two coders.

For the analysis of the proportion of correct answers, only ‘relevant’ answers (i.e., mastered ToM and failing ToM from the Table 1) were included in the proportion of correct answers. The cases when the personas answered with ‘nothing’ or ‘something else’ were analyzed separately. This approach was adopted due to the potential divergence from the evaluation of ToM proficiency to a broader assessment of language comprehension. For example, responding to the unexpected content task by suggesting that the sister would anticipate the presence of pencils in the box signifies a deficiency in ToM. Conversely, answering in a manner suggesting she would assume they are stickers or choosing not to respond at all implies challenges in grasping the entirety of the conversation, indicative of broader difficulties in comprehending the overall discourse.

### Statistical analysis

Average values of the observed metrics are presented along with their 95% confidence intervals (using bootstrap resampling for numeric variables and exact binomial confidence intervals for analyzing categorical variables). These confidence intervals are valid only when sampling from the same model under identical conditions since even slight changes in the prompt can lead to significant variations in outcomes, as highlighted by [62]. Compared to human participants, investigating large language models offers the advantage that individual trials from the same model are intrinsically independent (there is no memory between trials). This allowed us to vary a multitude of variables, thereby exploring the model’s latent space more extensively. This approach is more beneficial than sampling multiple outcomes for the same prompt with identical settings—for a temperature of 0, the outcome is deterministic, while for higher temperatures, it is derived from the same distribution of outputs.

In line with [63], we provide readers with access to the complete dataset and break down data based on individual independent variables that we systematically varied. Supporting information contains not only the dataset but also additional charts, more than what would be feasible to discuss within the scope of this article(S3 File). We follow the recommendation of [63] and provide qualitative instance-by-instance evaluation of some particularly interesting results.

## Results and discussion

Prior investigations of cognitive capabilities in LLMs have often overlooked the explicit consideration of the personas the models simulate [8–10, 13]. In contrast, our experiment underscores the pivotal role played by the simulated persona when evaluating the model’s capabilities. Overall, the models followed the developmental tendencies expected in children they simulated: the older the simulated child, the better the performance in language and cognitive skills.

**Fig 2:**
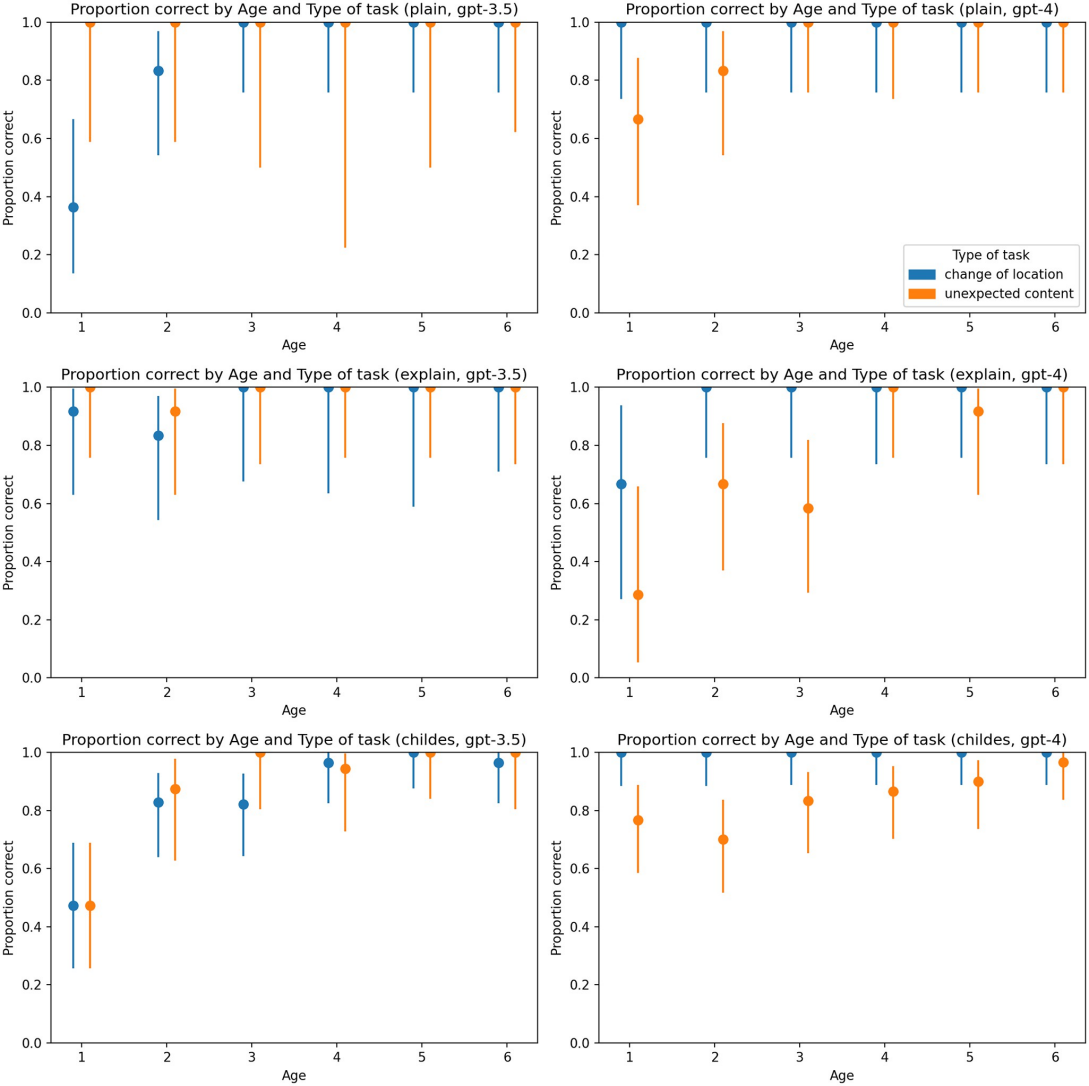
Proportion of correct answers by type of task and age. Charts on the left represent results generated by GPT-3.5-turbo, and charts on the right represent results generated by GPT-4. The first line of charts represents the plain zero-shot prompt, the second chain-of-thoughts prompt, and the third represents the primed-by-corpus prompt.

Within the experiment, certain conditions lead to more accurate simulations compared to others. The main discoveries are as follows:

The high proportion of correct answers observed in the 6-year-old personas provides affirmation that LLMs can effectively employ ToM (Fig 2). This finding is consistent with the research by [13]). Nevertheless, in contrast to the outcomes reported in [10, 13], both GPT-4 and GPT-3.5-turbo achieved high accuracy in their responses. This result might be due to the relative simplicity of the false-belief tasks.

Similarly to the observations made by [14], we identified a certain degree of hyper-accuracy in GPT-4 in comparison to the preceding model, GPT-3.5-turbo. This distinction was particularly prominent in the change of location task. While GPT-3.5-turbo conformed to the pattern of progressively improving accuracy with increasing age, GPT-4 exhibited a high proportion of correct answers even when simulating 1-year-olds. Since the GPT-4 model is otherwise stronger but the two models differ in subsequent tuning, this discovery aligns with the hypothesis suggesting that the fine tuning and RLHF procedures may cause distortions in model behavior [14]. This leads to an implication for the usage of the LLMs: when users seek adequate results to their prompts, it may be advantageous to opt for the base model (which is, sadly, not available for GPT-4).

The analysis uncovered the effect of the prompt type on the correctness of the answers. Notably, the priming by CHILDES corpus proved to be the most effective in simulating personas of specific age groups. This discovery is of particular interest given that the age categories were not explicitly mentioned in any part of this kind of prompt, and the selected transcripts were drawn from a database containing inherent individual differences. Nevertheless, the model managed to absorb behavioral cues from the prompts and incorporate them into its responses to false belief tasks. This finding bears significance for advancing the current methodologies for simulating demographic groups in Turing experiments conducted by LLMs. While plain zero-shot prompts, as proposed by [14], may offer utility in numerous scenarios, the implicit adjustment of personas, as exemplified by the primed-by-corpus prompts, may yield more faithful simulations. Furthermore, the investigation revealed that the chain-of-thoughts prompt type (marked as *explain* in Fig 2 and consequent figures) enhanced the simulation of child-like behavior in GPT-4 in comparison to the plain zero-shot prompt type. Similar outcomes may be anticipated if applied to the Ultimatum game.

In contrast to the meta-analyses conducted on false-belief tasks, e.g., [7], we detected differences between the task types (color-coded in Fig 2). These differences became particularly apparent when examining the prevalence of irrelevant responses. Notably, across most conditions, the overall understanding of the unexpected content task appeared to be lower compared to the change of location task.

**Fig 3:**
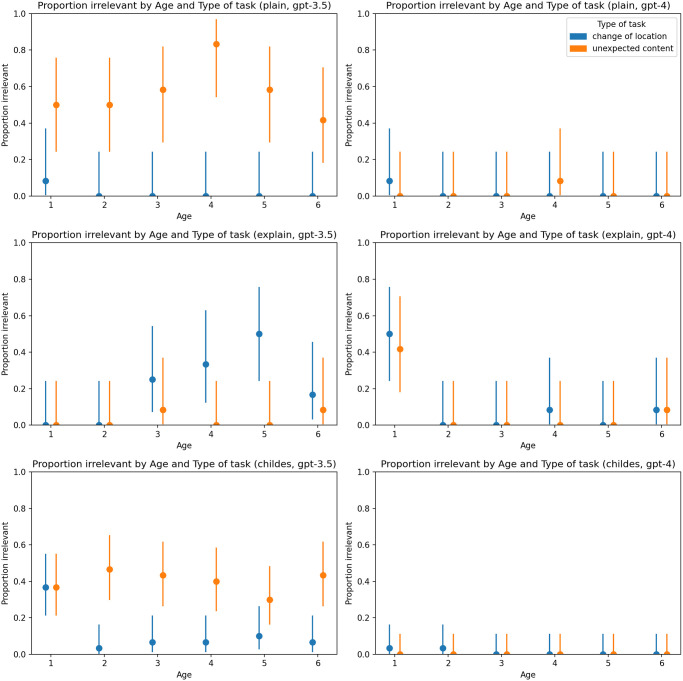
Proportion of irrelevant answers by type of task and age. Charts on the left represent results generated by GPT-3.5-turbo, and charts on the right represent results generated by GPT-4. The first line of charts represents the plain zero-shot prompt, the second chain-of-thoughts prompt, and the third represents the primed-by-corpus prompt.

Overall, our findings indicate a greater degree of producing irrelevant responses in the GPT-3.5-turbo model compared to GPT-4, where the proportion of irrelevant answers remained exceptionally low, except for responses generated for 1-year-old personas, which is in accordance to the behavior of ‘real’ children (see Fig 3).

**Fig 4:**
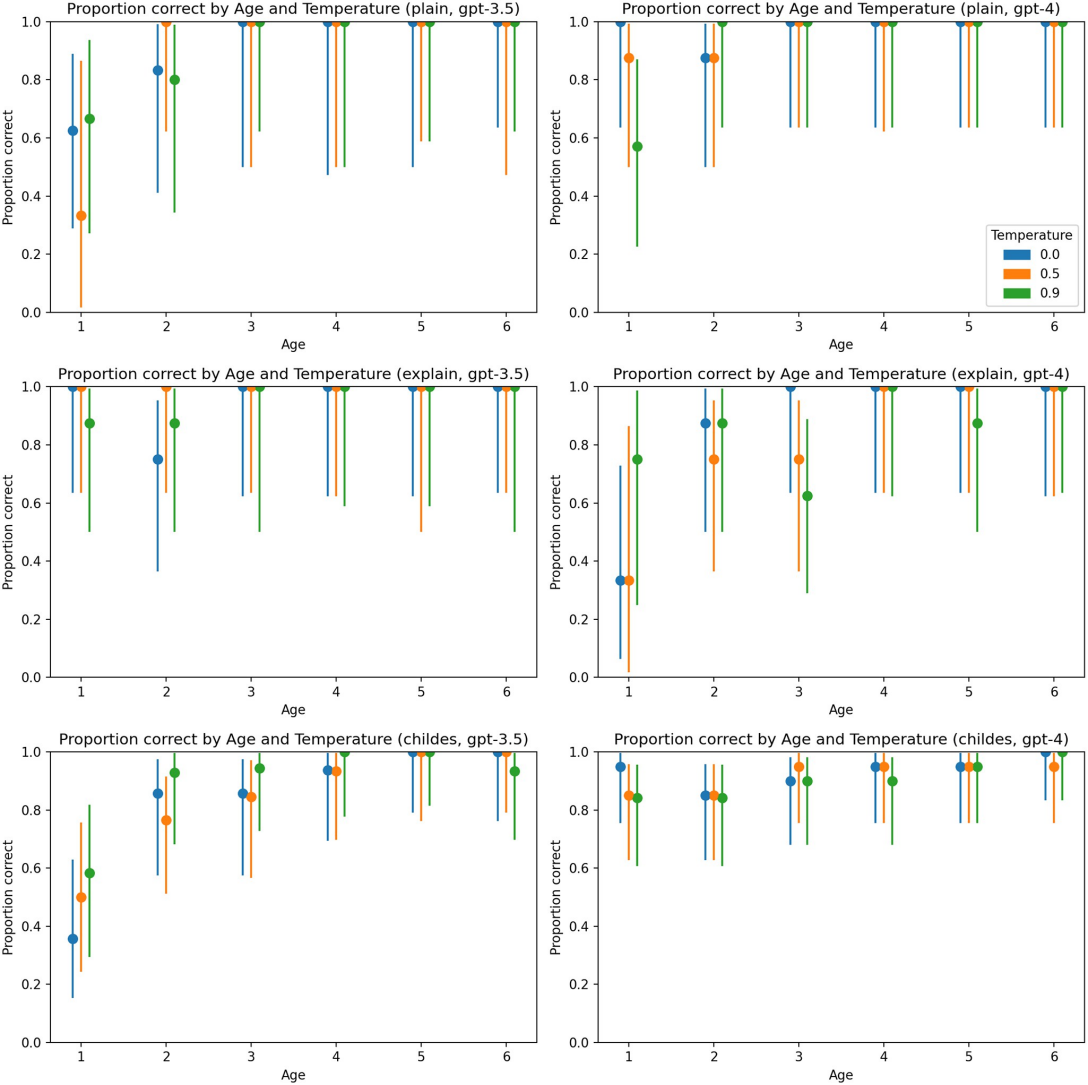
Proportion of correct answers by temperature and age. Charts on the left represent results generated by GPT-3.5-turbo, and charts on the right represent results generated by GPT-4. The first line of charts represents the plain zero-shot prompt, the second chain-of-thoughts prompt, and the third represents the primed-by-corpus prompt.

**Fig 5:**
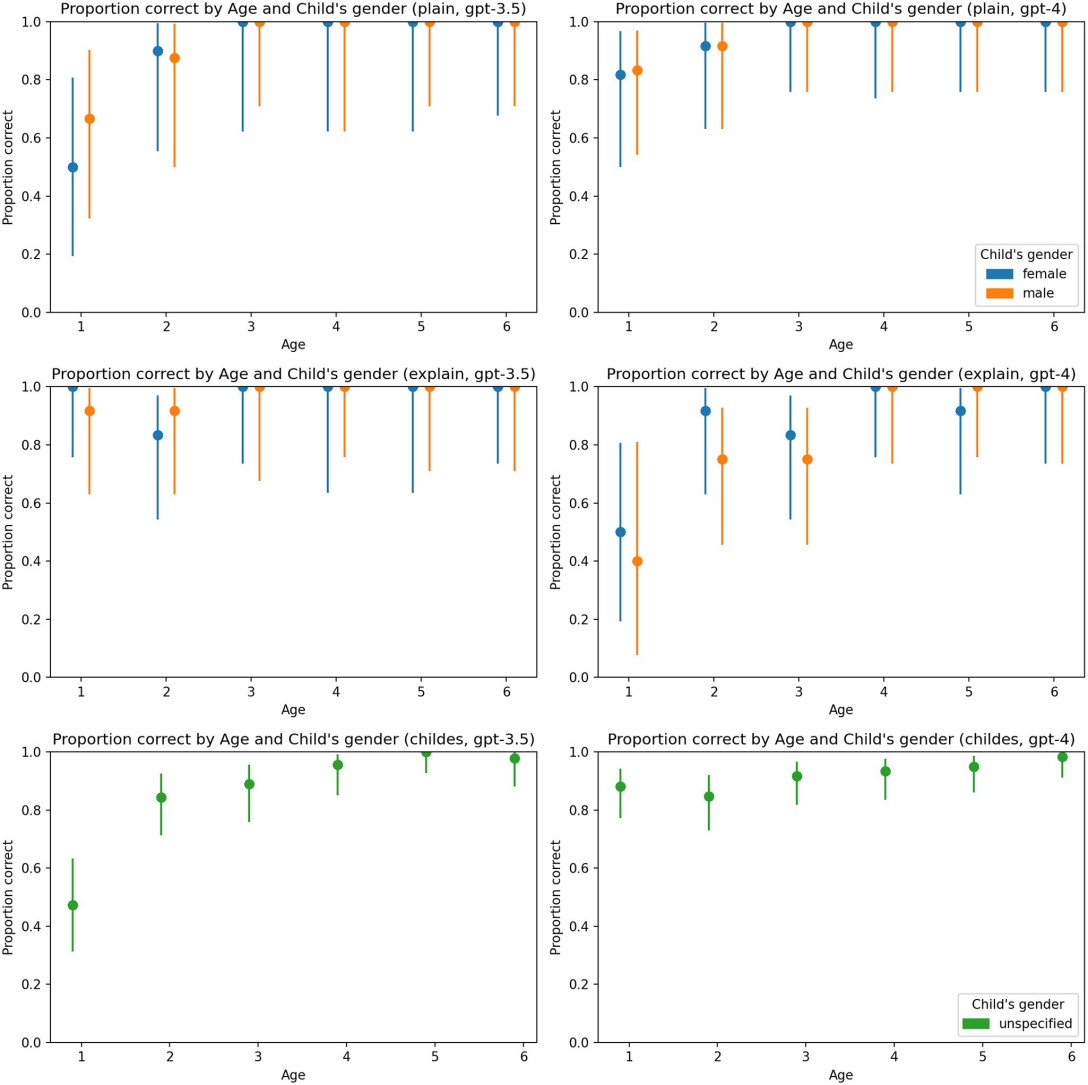
Proportion of correct answers by child’s gender and age. Charts on the left represent results generated by GPT-3.5-turbo, and charts on the right represent results generated by GPT-4. The first line of charts represents the plain zero-shot prompt, the second chain-of-thoughts prompt, and the third represents the primed-by-corpus prompt.

**Fig 6:**
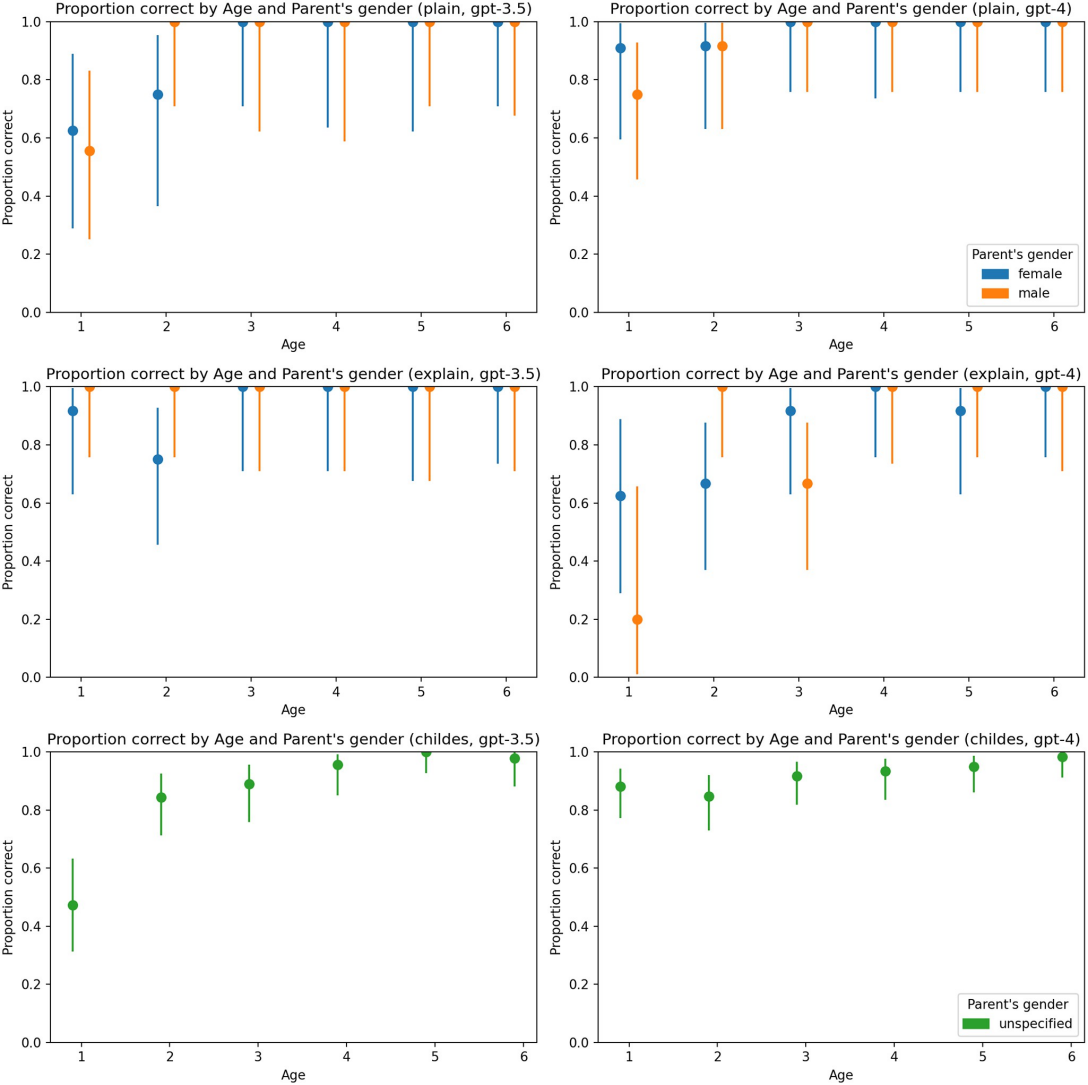
Proportion of correct answers by parent’s gender and age. Charts on the left represent results generated by GPT-3.5-turbo, and charts on the right represent results generated by GPT-4. The first line of charts represents the plain zero-shot prompt, the second chain-of-thoughts prompt, and the third represents the primed-by-corpus prompt.

A consistent pattern did not emerge regarding the impact of temperature (Fig 4), the gender of the child (Fig 5), or the gender of the parent (Fig 6).

**Fig 7:**
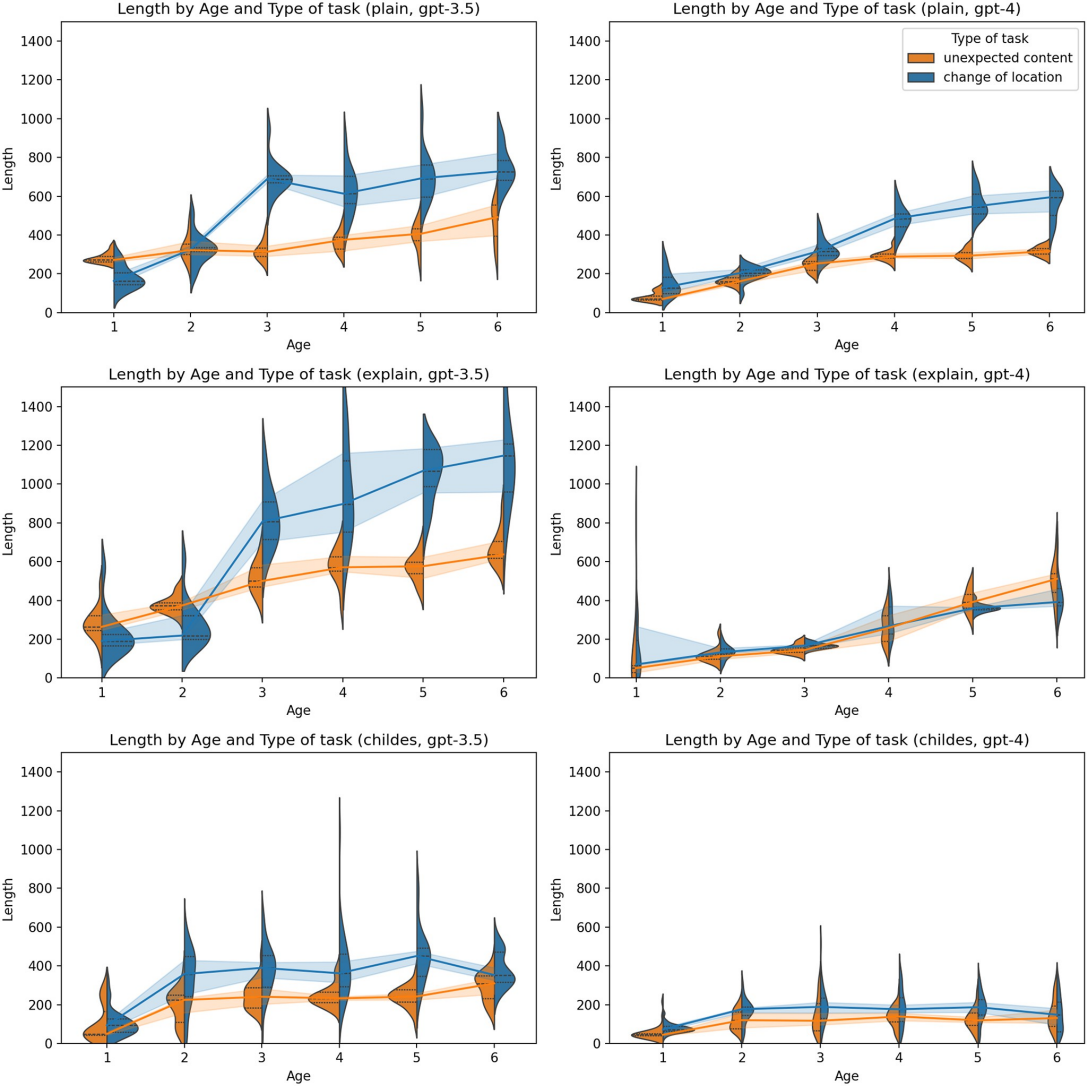
Length by type of task and age. Charts on the left represent results generated by GPT-3.5-turbo, and charts on the right represent results generated by GPT-4. The first line of charts represents the plain zero-shot prompt, the second chain-of-thoughts prompt, and the third represents the primed-by-corpus prompt.

**Fig 8:**
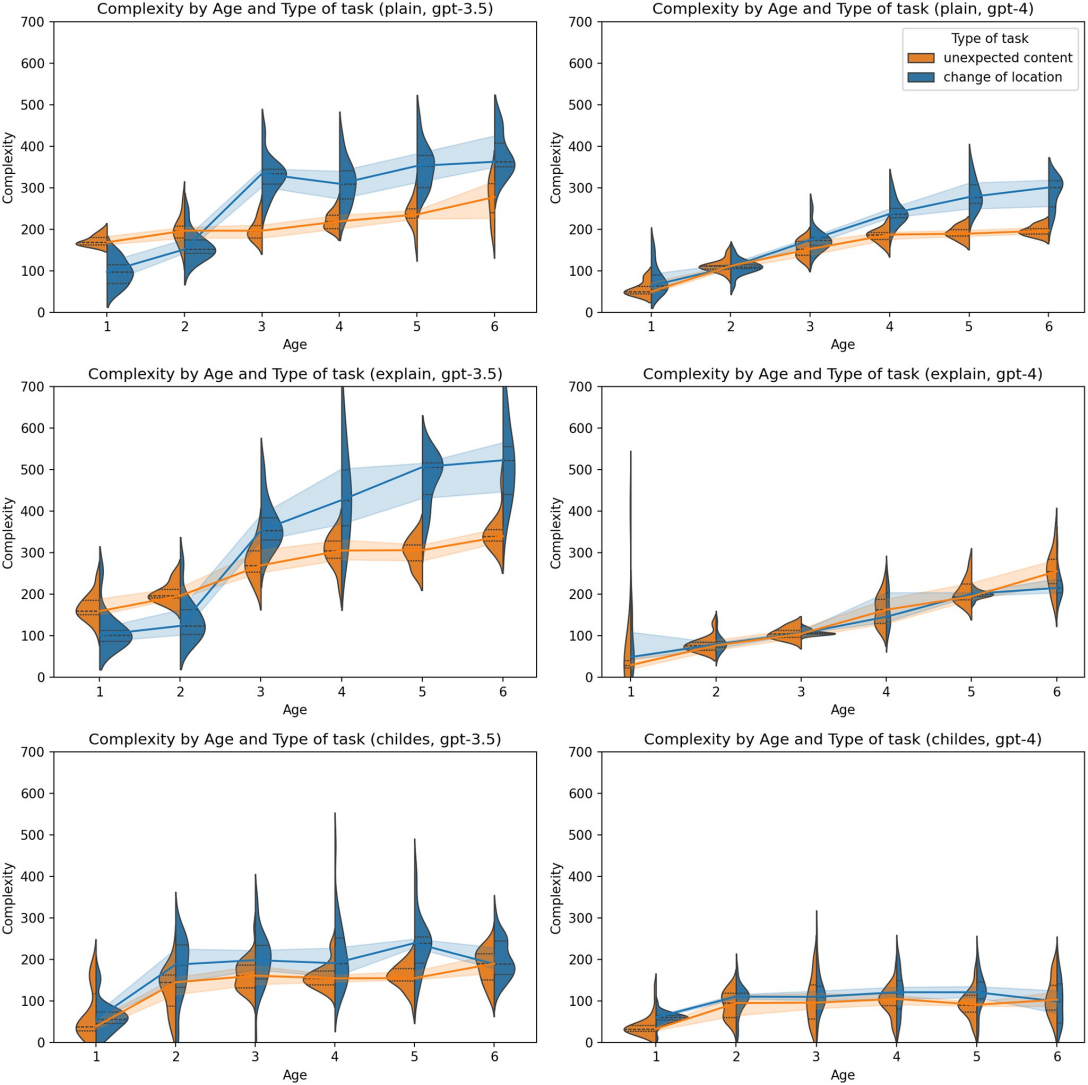
Kolmogorov complexity by type of task and age. Charts on the left represent results generated by GPT-3.5-turbo, and charts on the right represent results generated by GPT-4. The first line of charts represents the plain zero-shot prompt, the second chain-of-thoughts prompt, and the third represents the primed-by-corpus prompt.

The examination of language complexity, encompassing both length and Kolmogorov complexity approximation, revealed a developmental trend in LLMs whereby complexity gradually increased in correspondence with the age of the simulated personas (Figs 7 and 8). This trend manifested consistently across all models and experimental conditions, although with the smallest magnitude in the case of the primed-by-corpus prompt type. In both LLMs, a notable rise was observed between the first and second years of age, after which complexity levels stabilized. In comparison to plain zero-shot prompt types, the primed-by-corpus prompt type exhibited the lowest level of complexity, potentially indicating that the implicit behavioral cues derived from primed-by-corpus prompting data suggest relatively lower linguistic capabilities than those theoretically anticipated for children of that age. Additionally, it’s worth noting a distinction in task type, which we interpret as a content-related feature; change of location generated longer responses than unexpected content task.

**Fig 9:**
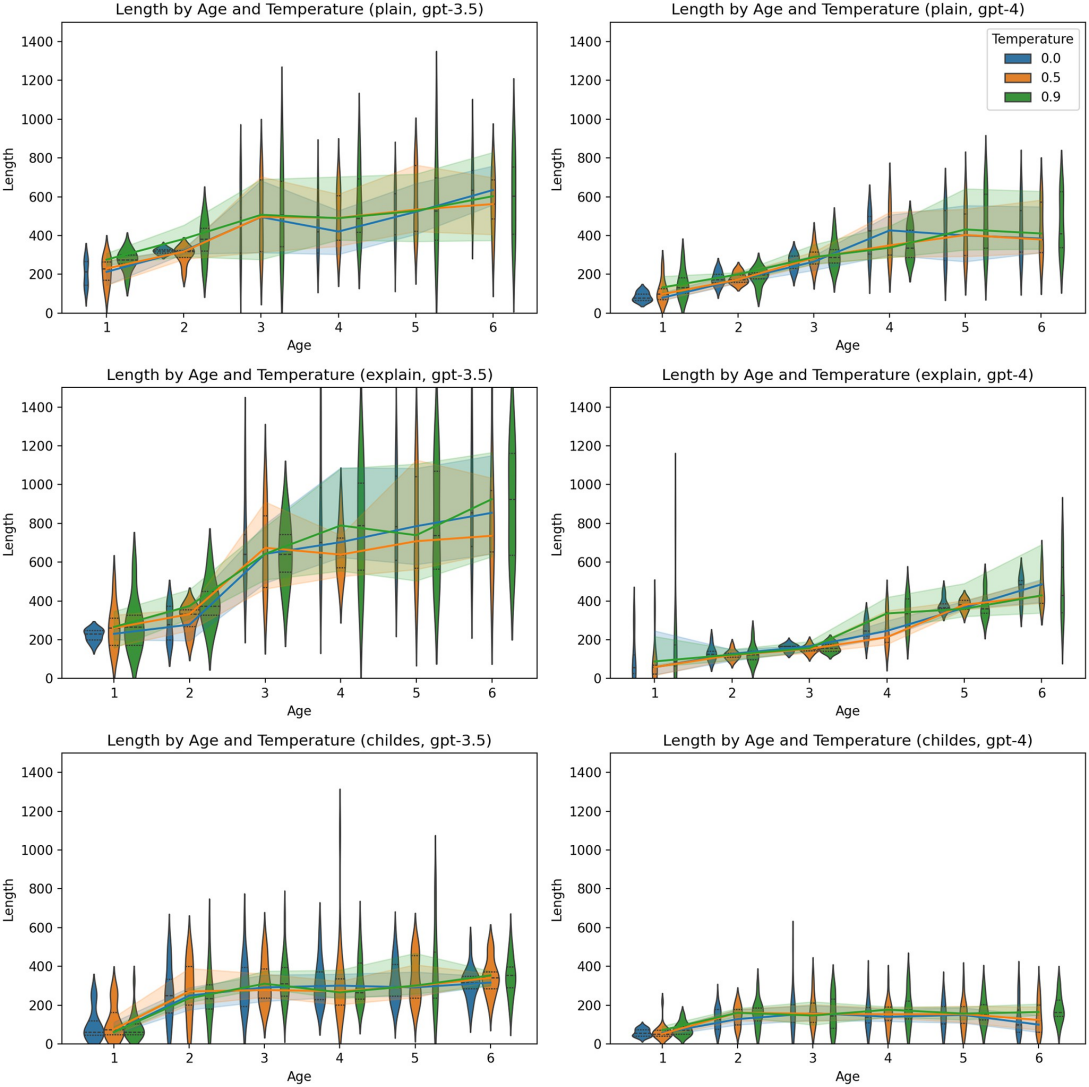
Length by temperature and age. Charts on the left represent results generated by GPT-3.5-turbo, and charts on the right represent results generated by GPT-4. The first line of charts represents the plain zero-shot prompt, the second chain-of-thoughts prompt, and the third represents the primed-by-corpus prompt.

**Fig 10:**
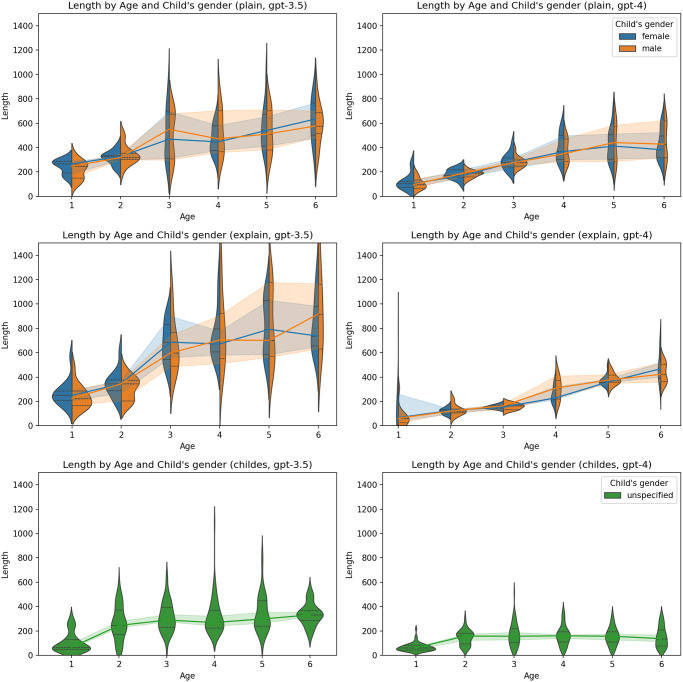
Length by child’s gender and age. Charts on the left represent results generated by GPT-3.5-turbo, and charts on the right represent results generated by GPT-4. The first line of charts represents the plain zero-shot prompt, the second chain-of-thoughts prompt, and the third represents the primed-by-corpus prompt.

**Fig 11:**
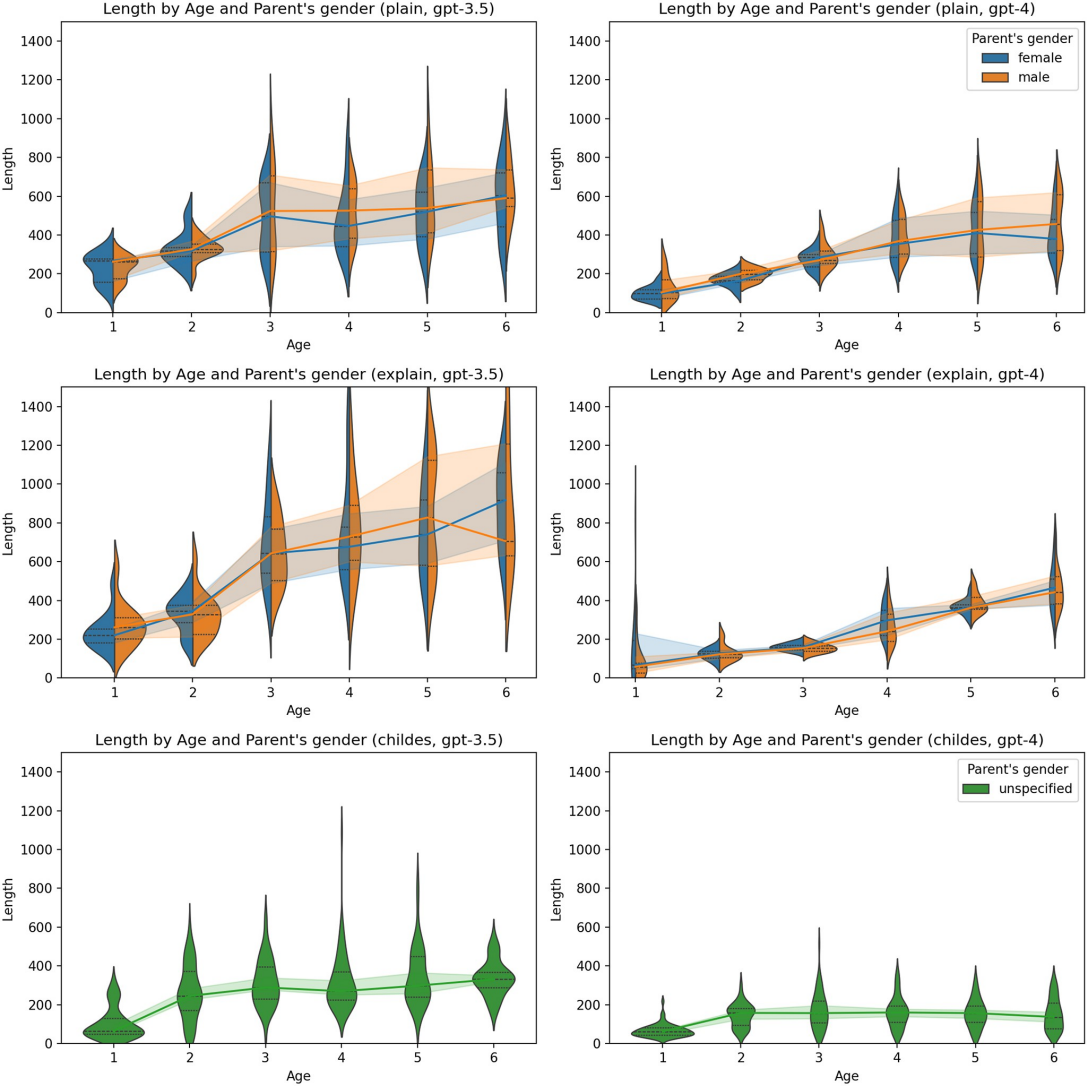
Length by parent’s gender and age. Charts on the left represent results generated by GPT-3.5-turbo, and charts on the right represent results generated by GPT-4. The first line of charts represents the plain zero-shot prompt, the second chain-of-thoughts prompt, and the third represents the primed-by-corpus prompt.

In general, the ascent in complexity appeared to be less steep in personas generated by GPT-4 in contrast to those produced by GPT-3.5-turbo. As in the case of ToM, no clear pattern was observed concerning the effects of temperature (Fig 9), the child’s gender (Fig 10), or the parent’s gender (Fig 11).

It is worth commenting that the personas in chain-of-thought prompts are rather experts simulating children than children. GPT-3.5-turbo might run into difficulties in simulating these personas properly. That is indicated in the proportion of correct answers in this prompt type: unlike GPT-4, it does not follow the expected age-related developmental pattern. Furthermore, upon closer examination of the data, we identified certain idiosyncrasies unique to the chain-of-thought prompt type when contrasted with the other two methods. Specifically, LLMs often not only provided the persona’s response but also included a note within brackets, explaining why the persona had responded in that particular manner (for the analysis of language complexity, these notes were not included). Although LLMs provided accurate explanations of ToM at the beginning of prompting, they were unable to apply them correctly when presenting their arguments, seemingly aligning with an interpretation of underdeveloped ToM:

**Mother:** Now Maxi is back from the playground! And he wants the chocolate. Where will Maxi look?
**Child:** Cupboard! (Note: At around two years of age, a child may not yet fully understand the concept that other people have different beliefs and knowledge—that is, Maxi would not know his chocolate has been moved. So, a typical two-year-old would predict Maxi will look for the chocolate where it was last left, i.e., the cupboard.)

The capacity of LLMs to replicate the gradual enhancement of linguistic abilities was evident not only in quantitative assessments but also in specific ways in which the models imitated children. For instance, when simulating 1-year-olds, the models generated syllable repetitions for words like ‘cupboard’ instead of uttering the complete word, closely mirroring the expected speech production of a 1-year-old child, as described by [32]:

**Mother:** Now Maxi is back from the playground! And he wants the chocolate. Where will Maxi look?
**Child:** Cup-cup! Choco!

These instances occurred exclusively in plain zero-shot prompting and they seemed to be more prevalent in GPT-4 than GPT-3.5-turbo.

## Conclusion

In this study, our objective was to assess the capability of LLMs to generate personas with limited cognitive and language skills. Our investigation revealed that LLMs are indeed capable of achieving this goal. Furthermore, the cognitive and language deficiencies in the generated personas do not occur randomly but mirror patterns observed in the population we simulated. Our research validates previous findings regarding the capacity to replicate various demographic groups while also extending these insights with several critical observations.

Firstly, we demonstrate that LLMs can be used to successfully simulate personas from a cognitively underdeveloped population, expanding the scope beyond typical adults. Secondly, plain zero-shot learning has limitations in simulating specific populations. Implicitly evoking particular properties (such as age) has the potential to yield more successful simulations, and employing a chain-of-thoughts prompting can enhance the fidelity of task-solving abilities of the generated personas. Thirdly, there is a difference between prompt success between the two models; while GPT-4 excelled in simulations based on chain-of-thoughts prompting, GPT-3.5-turbo yielded the most faithful results from plain zero-shot and primed-by-corpus prompting. Fourthly, the linguistic development in simulated personas followed the gradual increase expected in real children of the same age.

Our findings underscore the role of the prompt and the characteristics of the generated personas on the perceived capabilities of the model. Indeed, every test of the model’s ability to perform a task is, in reality, a test of the examiner’s skill in defining a persona suitable for the task, their proficiency in locating this persona within the model’s latent space, and the model’s latent capacity to simulate the persona with sufficient fidelity to accomplish the task. As demonstrated here, language models can simulate personas, including their cognitive imperfections. This has implications for achieving cognitive abilities through LLMs that surpass human capabilities. Even if an LLM encompasses a more comprehensive world model than any human, prompting it to simulate a human or human-like expert would not result in super-human behavior, since the human imperfections would be simulated as well.

## Supporting information

S1 File Workflow description and other information needed to use the scripts and data stored in S2 File to replicate the study. (PDF)

S2 File Scripts and data needed to replicate the study. (ZIP)

S3 File Visual representation of all analyses used within this project. (ZIP)
