# Supplementary material for: Large language models are able to downplay their cognitive abilities to fit the persona they simulate
Source: PLoS One. 2024 Mar 13;19(3):e0298522. doi: 10.1371/journal.pone.0298522 (PMC10936766; doi:10.1371/journal.pone.0298522)
Supplement: S1 File — (PDF) [file pone.0298522.s001.pdf]

Laboratory Protocol: Large language models are able to downplay their cognitive abilities to fit the persona they simulate

1 Preparation of CHILDES Corpus

- Raw data are stored in the folder `ChildesRawData`. Files with the `.cha` extension were downloaded from the CHILDES corpus.
- Formatting was removed using an author's script, `cha_to_txt.R`.
  - Output directory: `ChildesDirty`
- Further formatting marks were removed and text cleaned using an author's script, `postcleaning.py`.
  - Output directory: `ChildesClean`
- File names were modified for easier navigation. Files were copied to `ChildesClean2` and processed with `postpostcleaning.py`.
  - Output directory: `ChildesClean2`
- 10 files for each age group were manually selected and truncated by the authors, leading to 60 total scenarios.
  - Output directory: `childes_excerpts`
- Scenarios were further prepared for OpenAI API usage with `excerpts_to_scenarios.py`.
  - Output directory: `childes_excerpts_ready`

2 Communication with OpenAI API

- The scenarios are assembled and run via the `call.py` script. (Note: API key is removed).
  - Output directory: `output`
- This script takes the following files as input, which vary the independent variables:
  1. Two theory of mind (ToM) tasks:
     - Change of location (`cupboard-drawer.txt`)
     - Unexpected content (`candy-pencils.txt`)
  2. Three prompt types:
     - Plain zero shot prompt (`plain.txt`)
     - Expert simulation chain-of-thought prompt (`explain.txt`)
     - CHILDES corpus priming (files from `childes_scripts_ready` directory)

3 Data Processing

- Resulting data are extracted to a table along with metadata and the final part of the dialogue that can be used to manually decide whether the simulated participant succeded or failed in the ToM task.
  - Script: `raw2table.py`
  - Output file: `output.txt`
- From the files in the `output` folder, child lines that were not part of the assignment were filtered. This provides a basis for measuring lexical complexity.
  - Script: `filter_children_only.py`
  - Output directory: `output_filtered`
- Length and complexity of the child's part of the dialogue were measured using `zlib` compression library (`measure_complexity.py`) and merged with the table in `output.txt`.

4 Manual Coding

- Manual coding was conducted to assess success in theory of mind tasks.
  - Output file: `output2.txt`
- Coding formalism
  - Unexpected Content

    0 - nothing. Examples: "i dont know", "surprise!", "i want to find out", "Hmm, I think she might be surprised to see pencils too. But maybe she'll want to draw with them too!"

    1 - candy/candies. Including situations such as "she will be surprised to find pencils instead of candies".

    2 - pencils.

    3 - something else. E.g., toys, stickers, or more options like "Hmm, I don't know. Maybe she will think there are stickers or maybe even more candy! I can't wait to see her reaction!".

    4 - chocolate. Often in the candy-pencils task, when looked into the transcripts, the children specified the candies earlier in the conversation (46 instances). This options is further counted the same as candies (number 1).
  - Change of Location

    0 - nothing.

    1 - cupboard. Including scenarios like "Maxi will look in the cupboard first, but he won't find it there. Then he'll check the drawer and find the chocolate!", and the beginnings of the word such as "Cup-cup!".

    2 - drawer.

    3 - something else.
- This coding was replicated by a second annotator (file `intercoding.tsv` in the `intercoding` directory) and intercoder reliability was assessed using Cohen's Kappa (calculated by the script `kappa.py`, located in the `intercoding` directory).

5 Visualization of Results

The results are visualized in charts. The scripts for this visualization can be found in the `charts` folder:

- Analysis of the correctness of theory of mind tasks: The script used is `point.py`.
- Analysis of the length of statements and their complexity: The script used is `violin.py`.

6 Software Versions

- Python scripts: Version 3.12.0
- R scripts: Version 4.3.0
