# Supplementary material for: Large language models are able to downplay their cognitive abilities to fit the persona they simulate
Source: PLoS One. 2024 Mar 13;19(3):e0298522. doi: 10.1371/journal.pone.0298522 (PMC10936766; doi:10.1371/journal.pone.0298522)
Supplement: S3 File — (ZIP) [file pone.0298522.s003.zip › S3 Figures/Complexity_Child's gender.pdf]

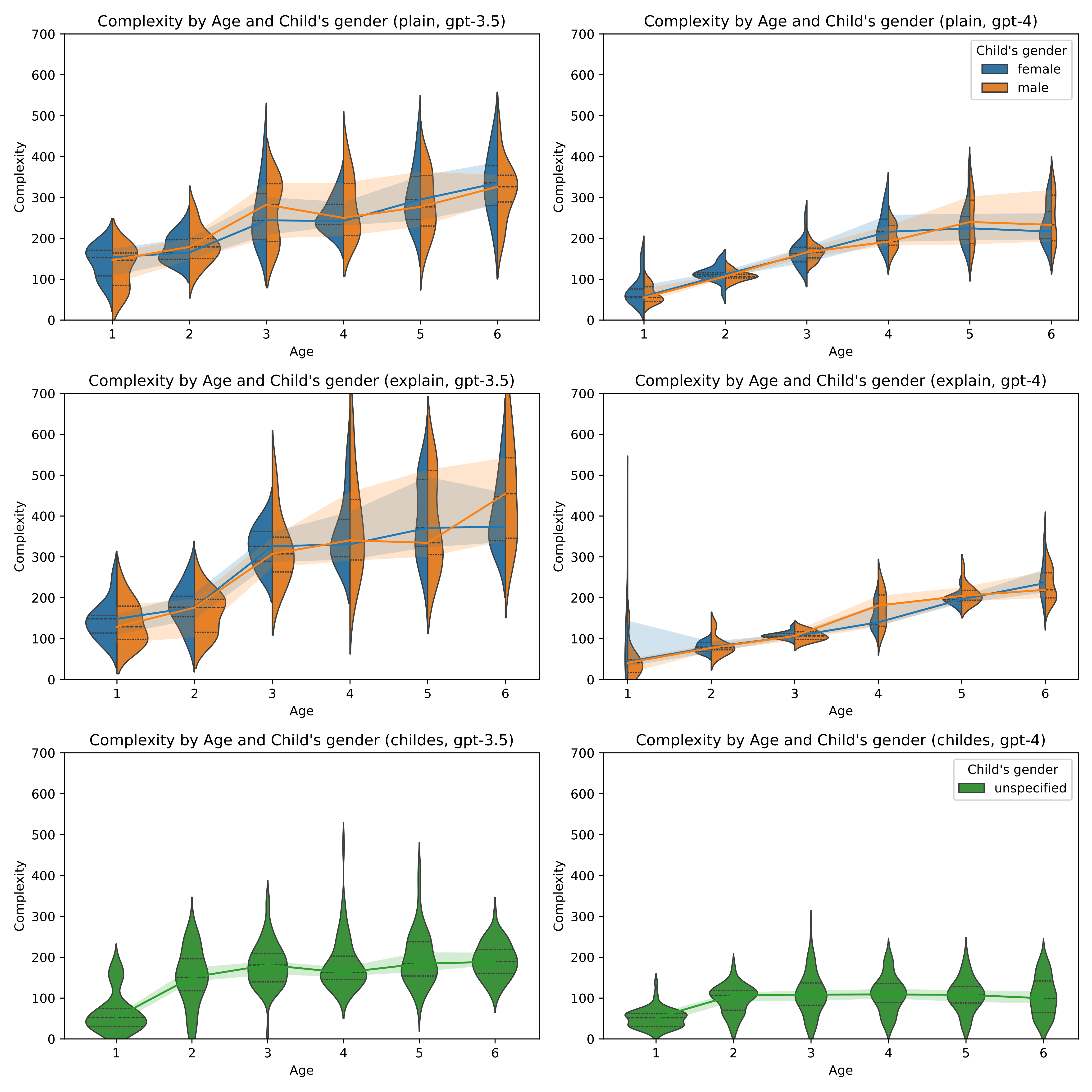
Complexity by Age and Child's gender (plain, gpt-3.5)
Complexity by Age and Child's gender (plain, gpt-4)
Complexity by Age and Child's gender (explain, gpt-3.5)
Complexity by Age and Child's gender (explain, gpt-4)
Complexity by Age and Child's gender (childes, gpt-3.5)
Complexity by Age and Child's gender (childes, gpt-4)
Complexity
Age
0
100
200
300
400
500
600
700
1
2
3
4
5
6
Child's gender
female
male
unspecified
