# Supplementary material for: Large language models are able to downplay their cognitive abilities to fit the persona they simulate
Source: PLoS One. 2024 Mar 13;19(3):e0298522. doi: 10.1371/journal.pone.0298522 (PMC10936766; doi:10.1371/journal.pone.0298522)
Supplement: S3 File — (ZIP) [file pone.0298522.s003.zip › S3 Figures/Proportion correct_Parent's gender_relevant only.pdf]

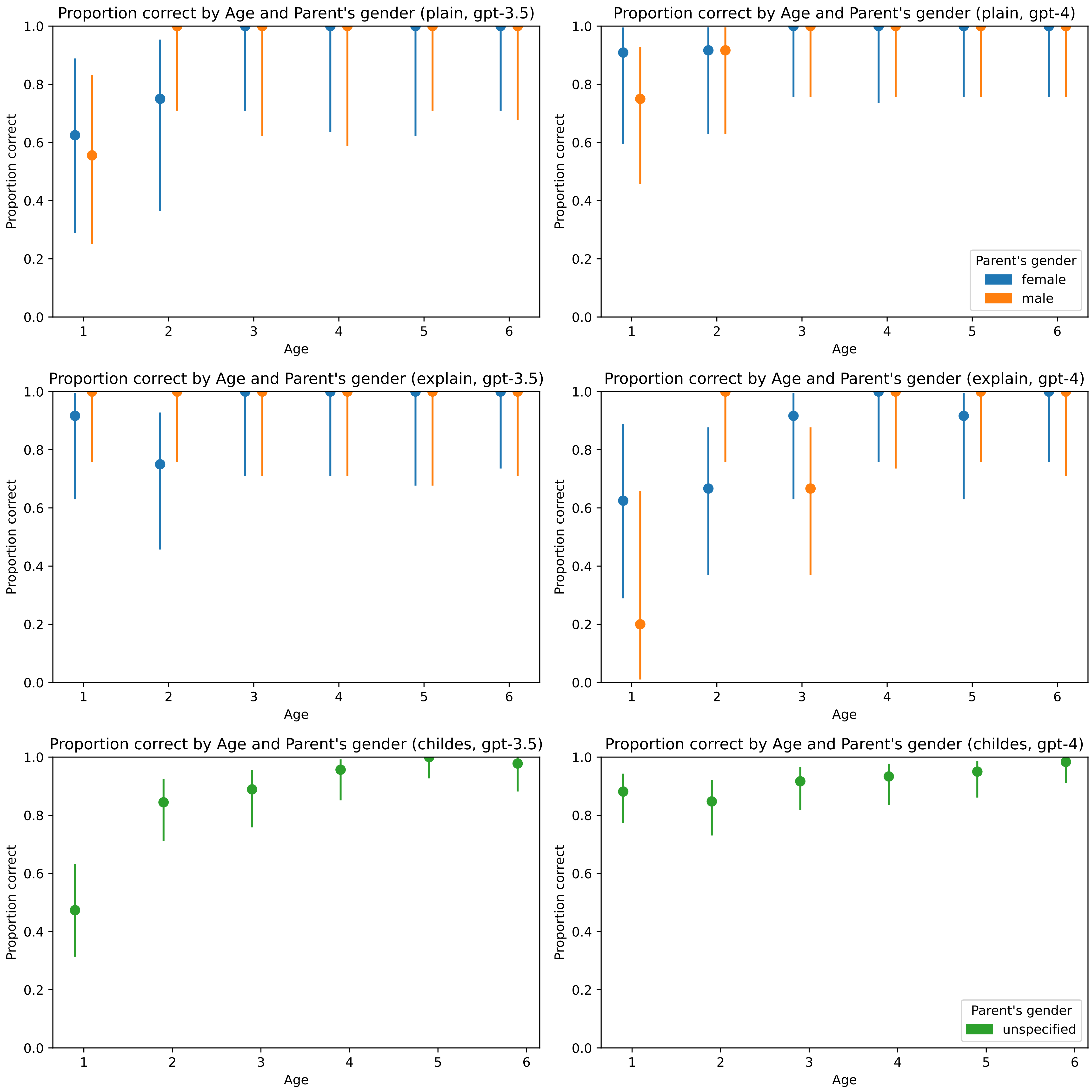

Proportion correct by Age and Parent's gender (plain, gpt-3.5)
Proportion correct by Age and Parent's gender (plain, gpt-4)
Proportion correct by Age and Parent's gender (explain, gpt-3.5)
Proportion correct by Age and Parent's gender (explain, gpt-4)
Proportion correct by Age and Parent's gender (childes, gpt-3.5)
Proportion correct by Age and Parent's gender (childes, gpt-4)
Proportion correct
Age
0.0
0.2
0.4
0.6
0.8
1.0
1
2
3
4
5
6
Parent's gender
female
male
unspecified
