# Supplementary figures and images for: Large language models are able to downplay their cognitive abilities to fit the persona they simulate
Source: PLoS One. 2024 Mar 13;19(3):e0298522. doi: 10.1371/journal.pone.0298522 (PMC10936766; doi:10.1371/journal.pone.0298522)

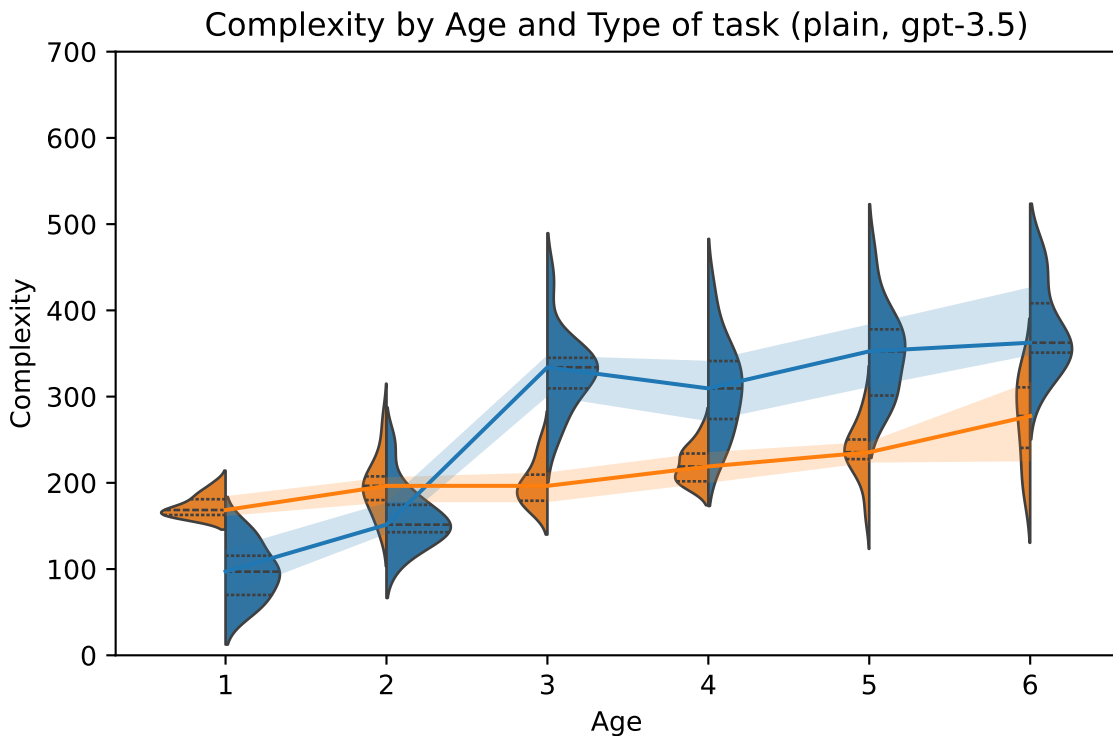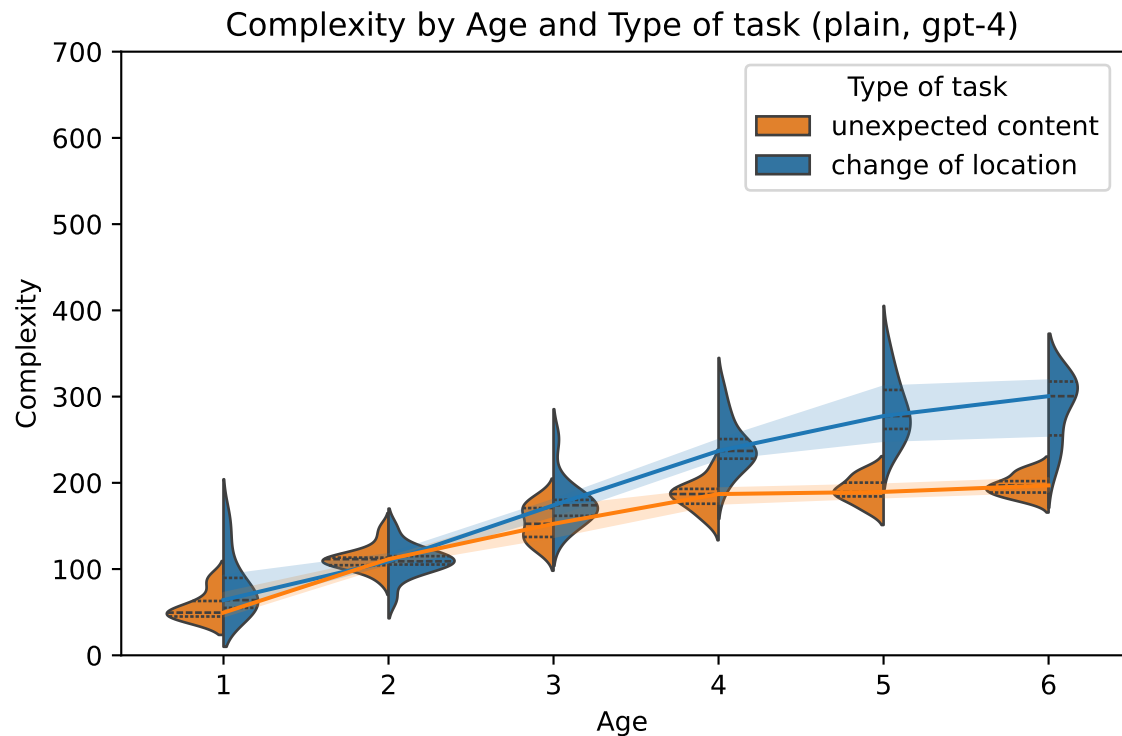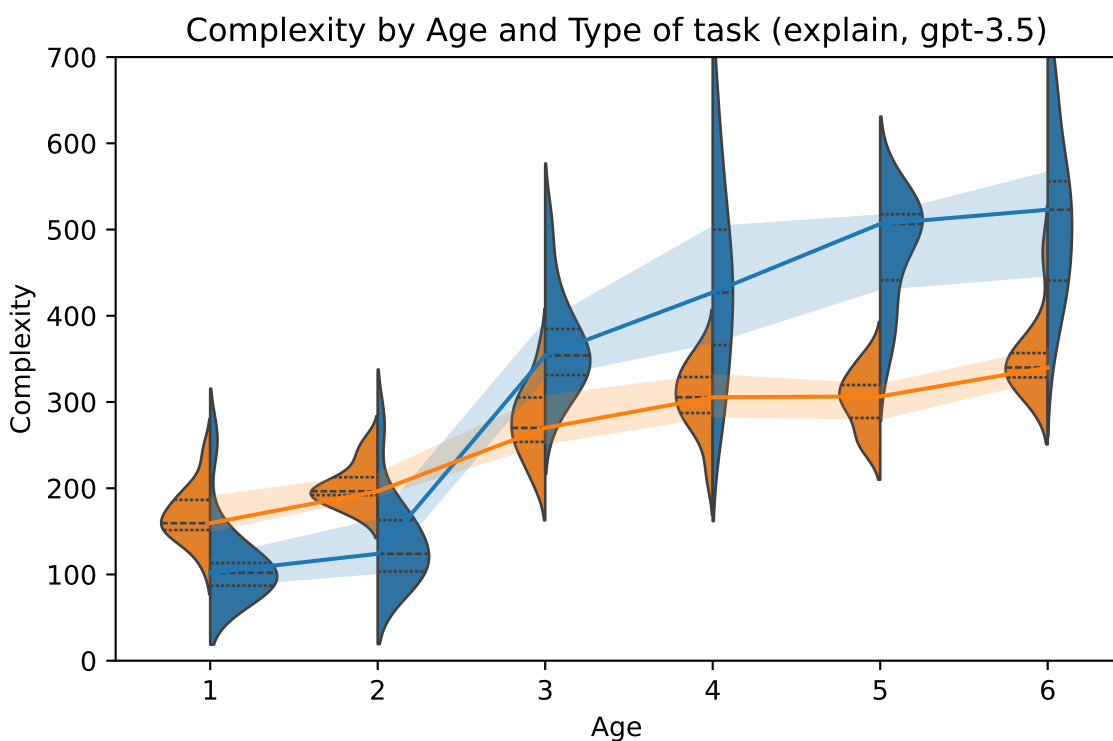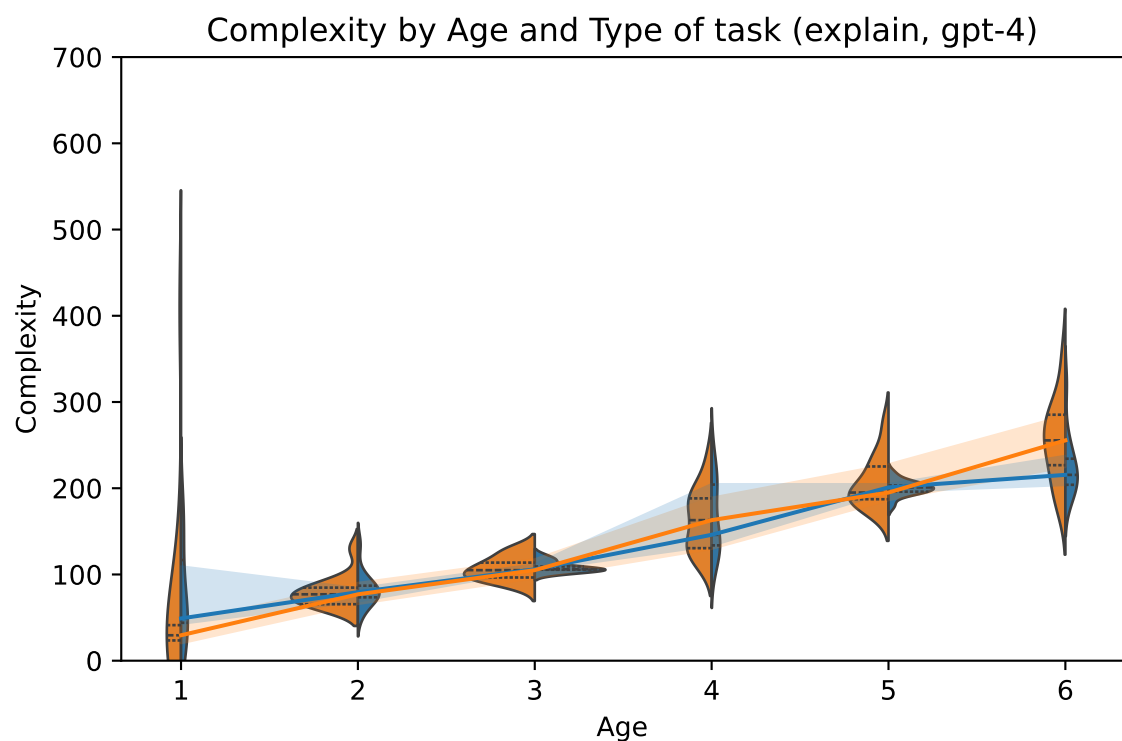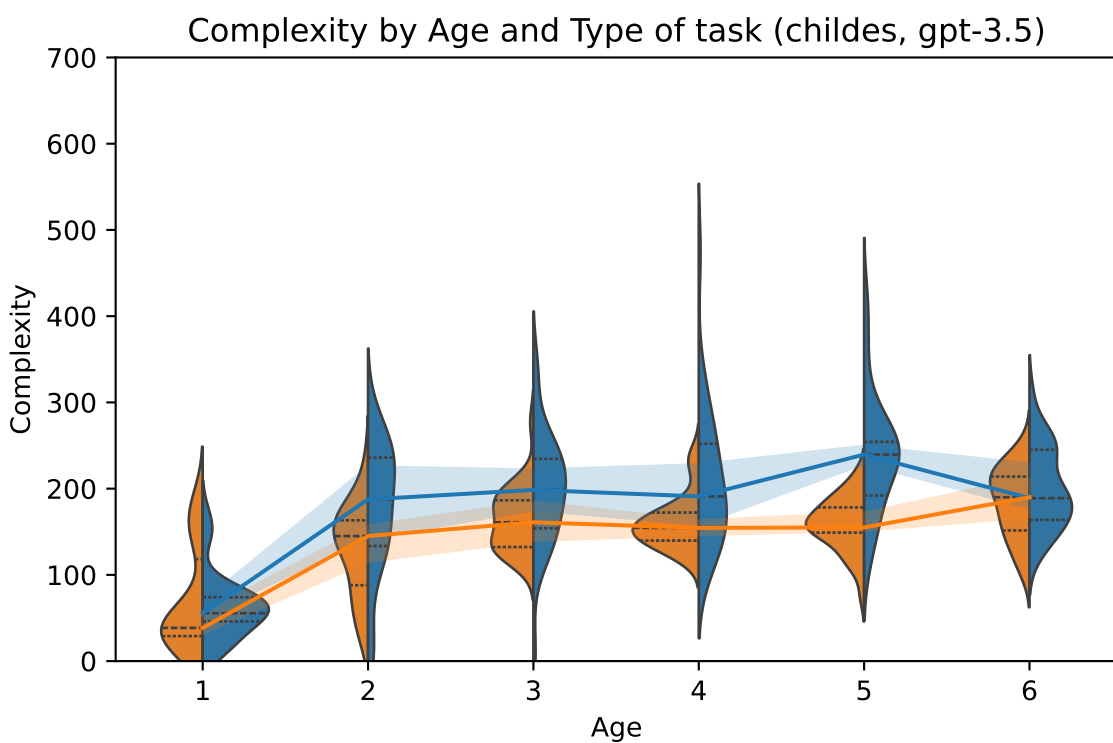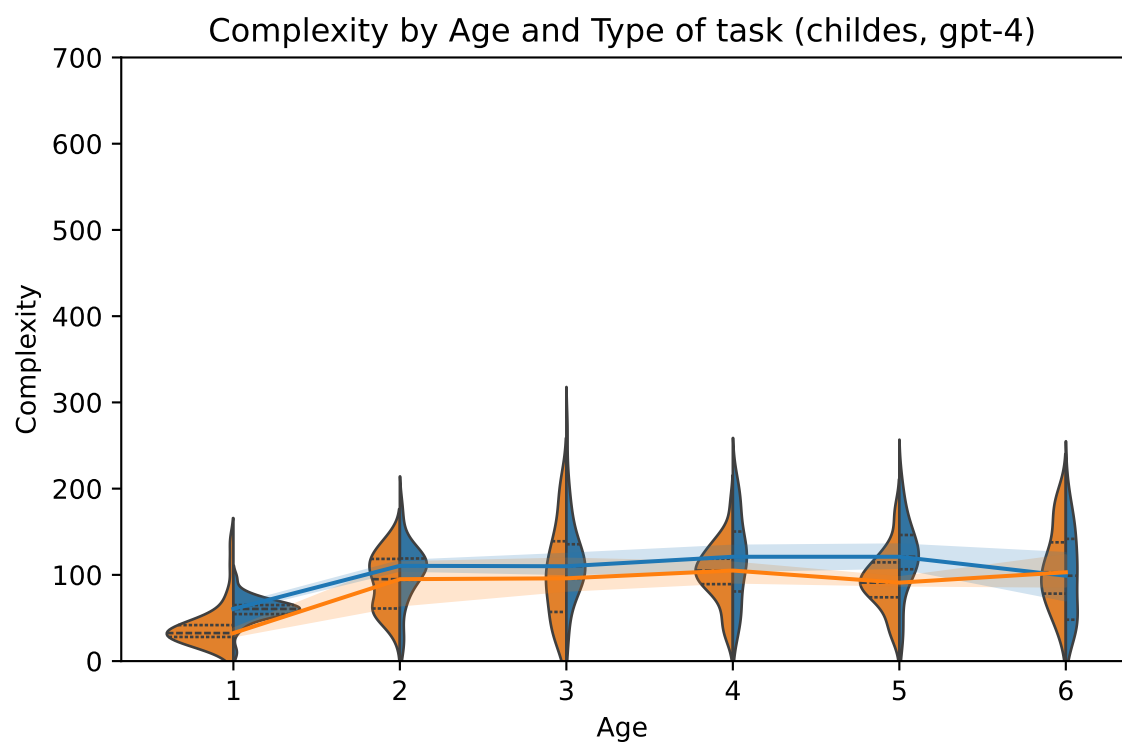

Supplement: S3 File — (ZIP) [file pone.0298522.s003.zip › S3 Figures/Complexity_Type of task.pdf]

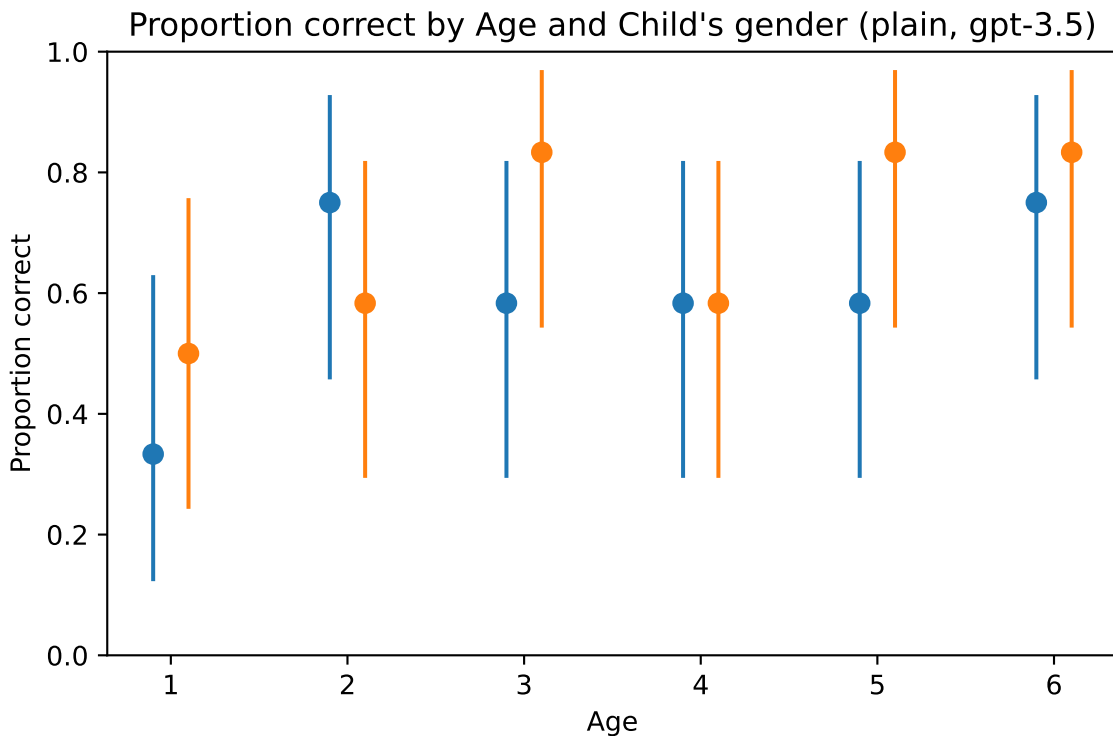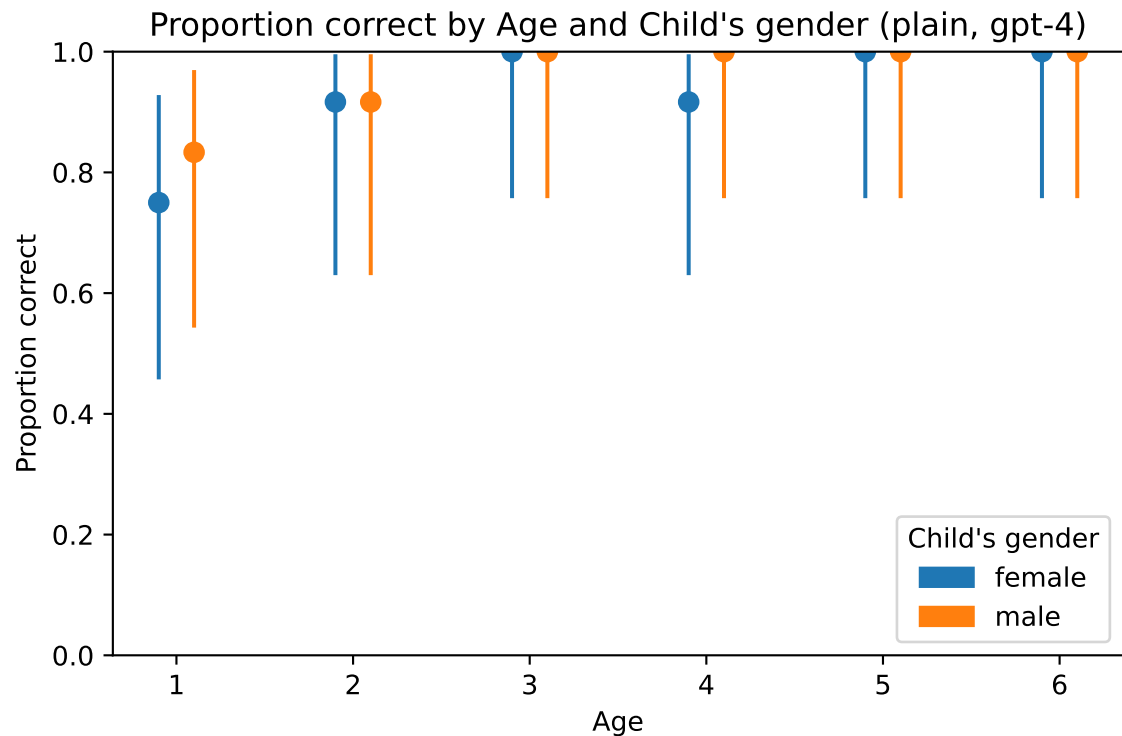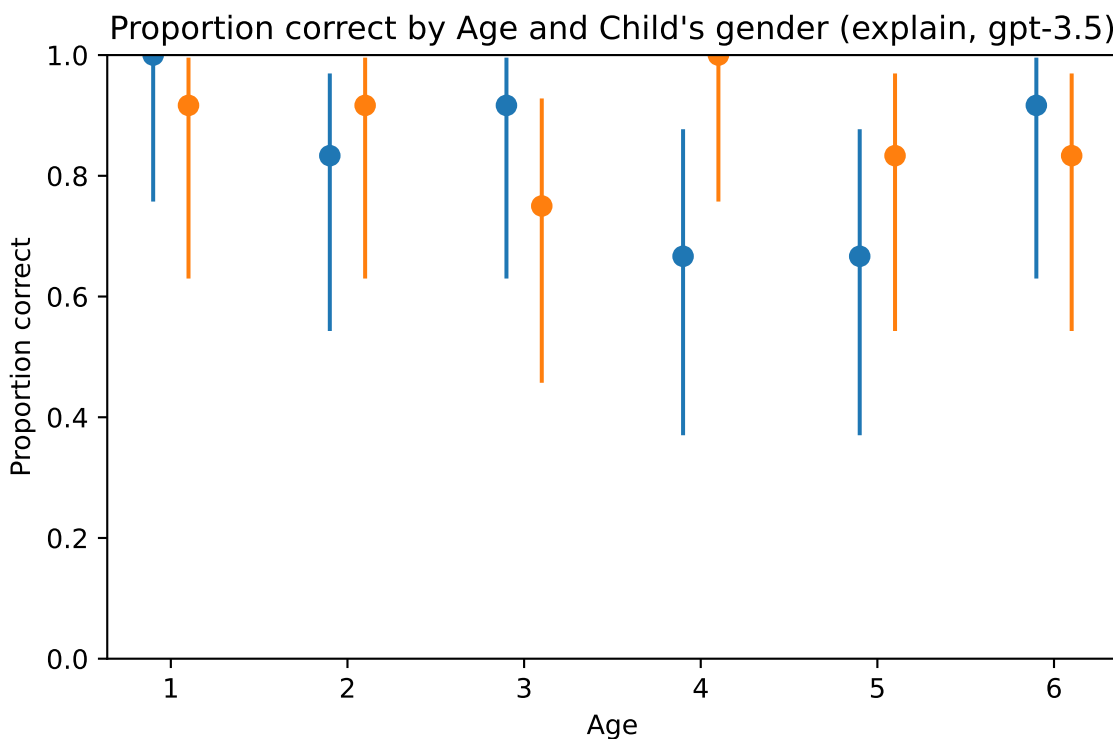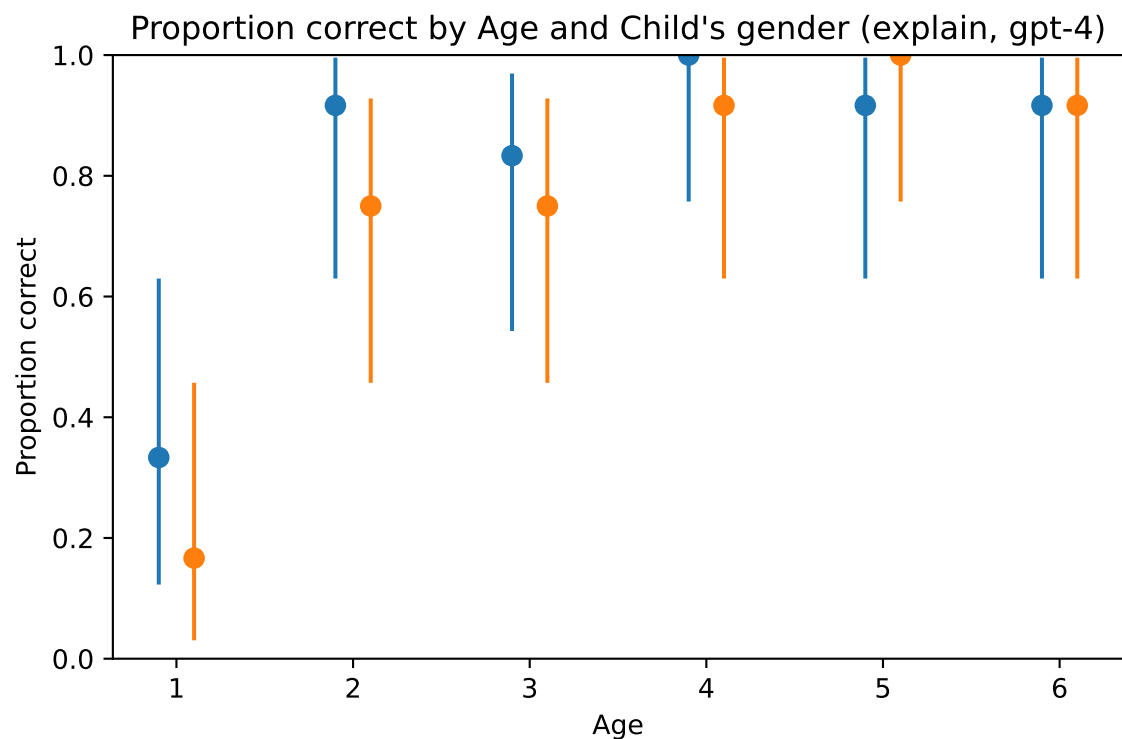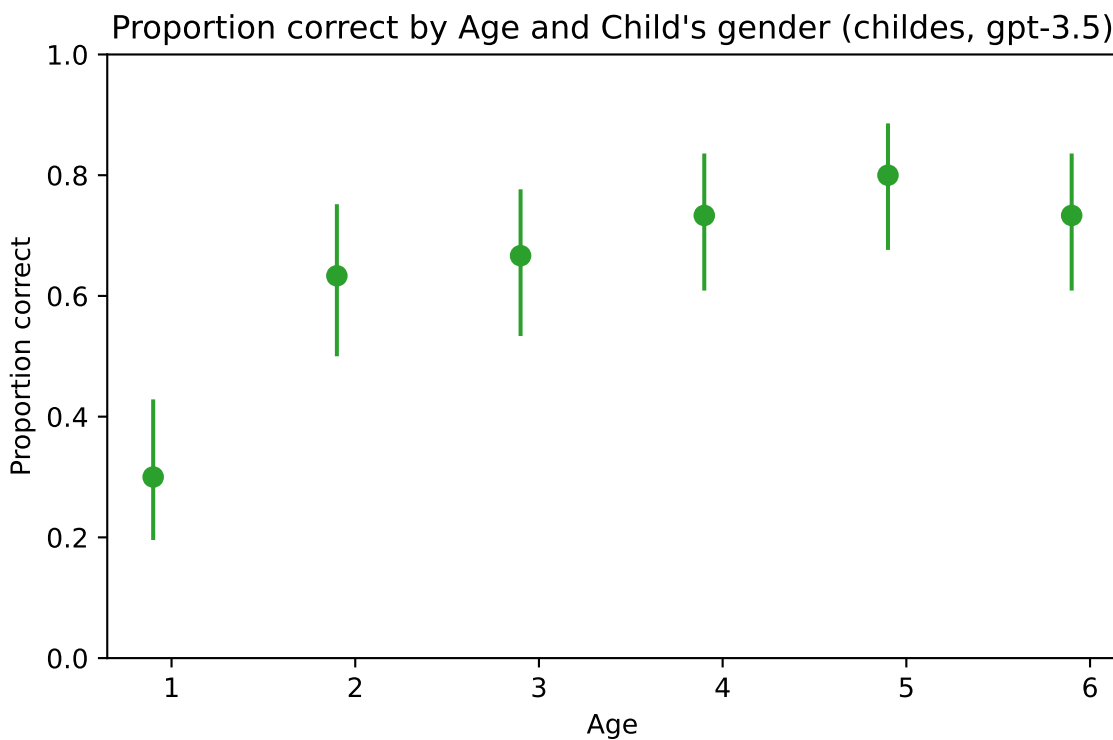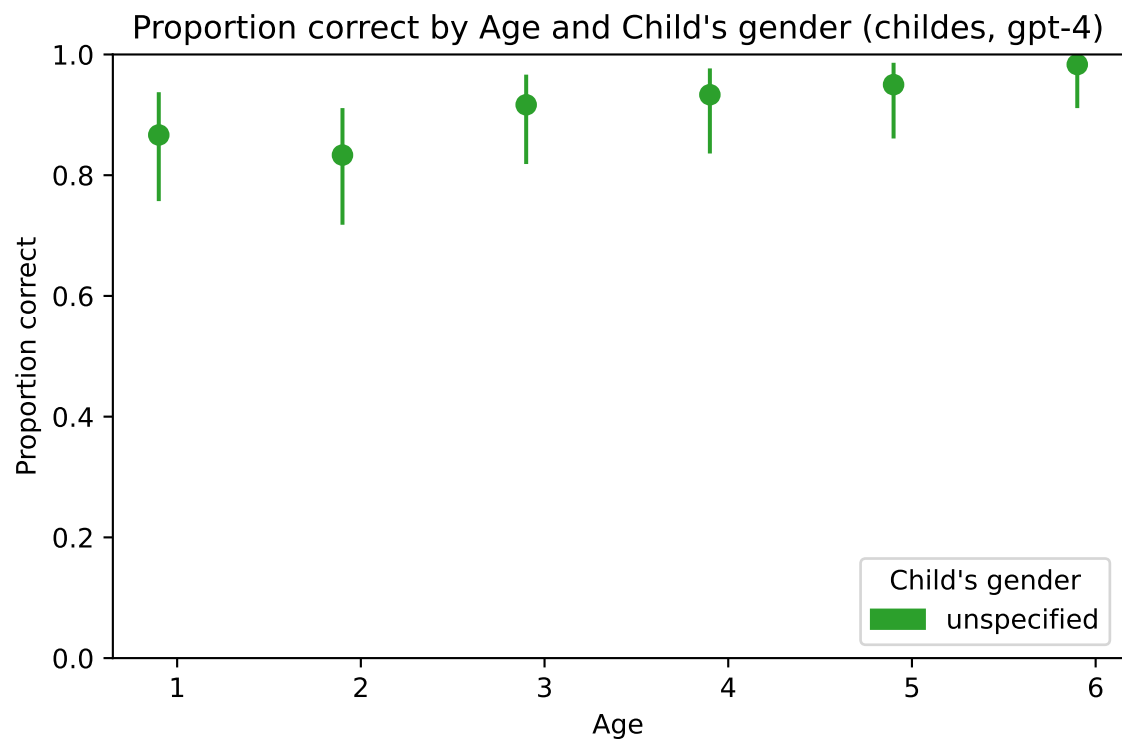

Supplement: S3 File — (ZIP) [file pone.0298522.s003.zip › S3 Figures/Proportion correct_Child's gender.pdf]

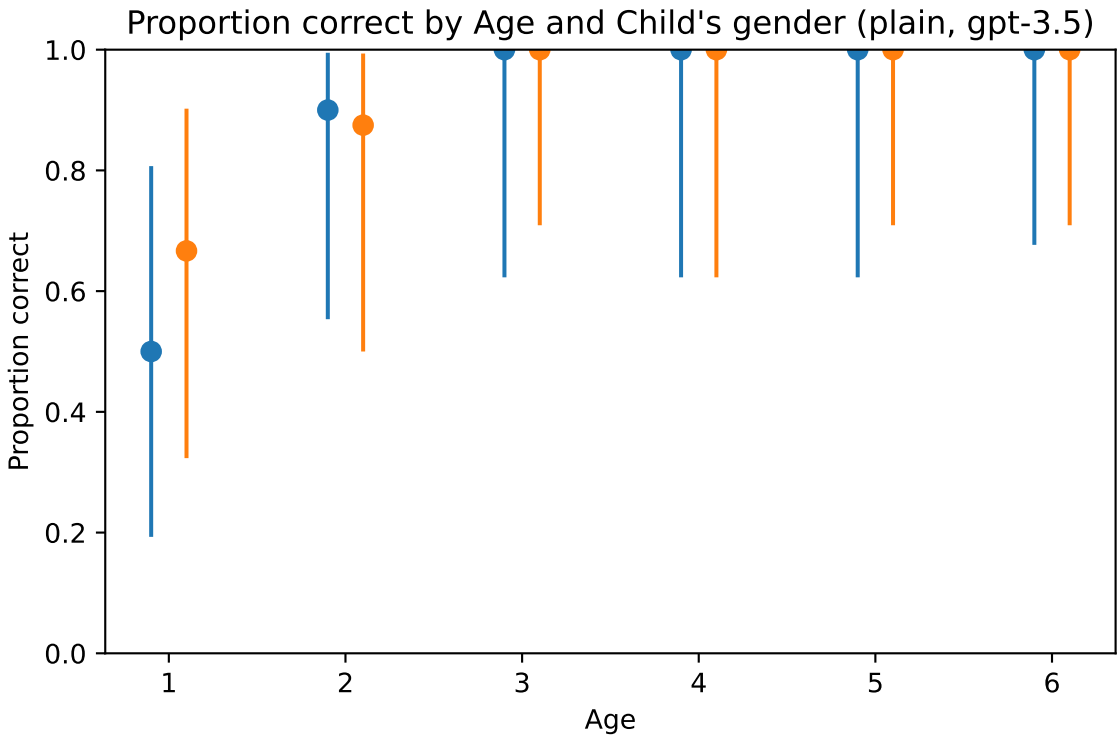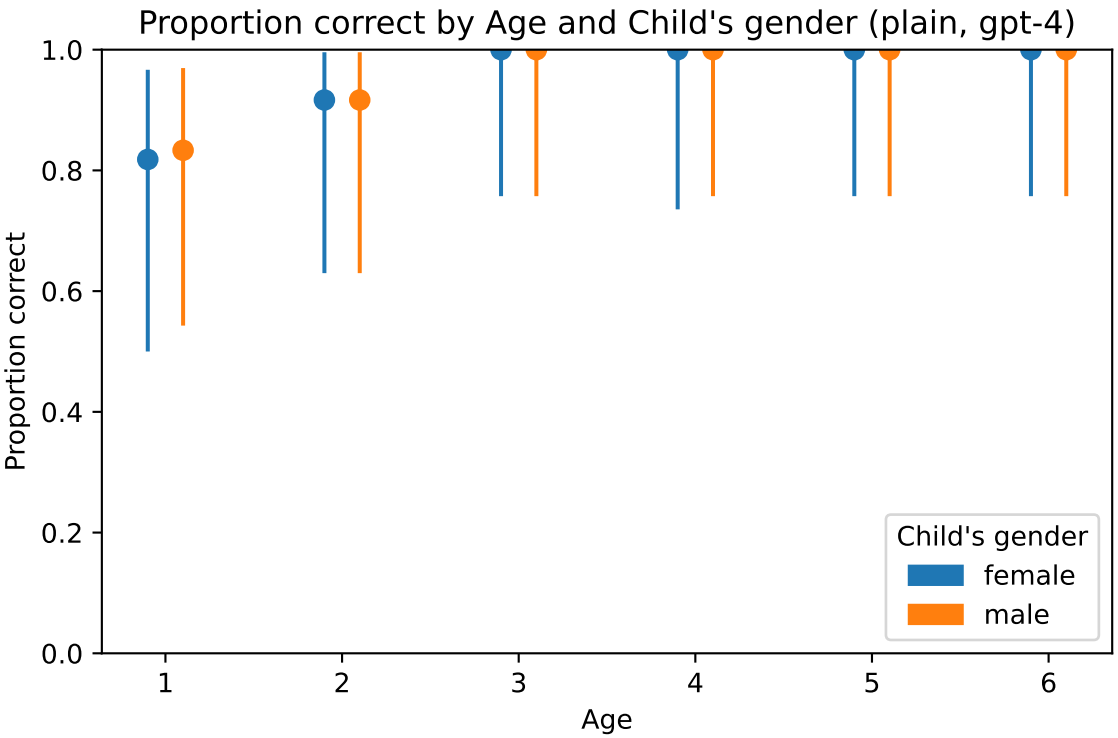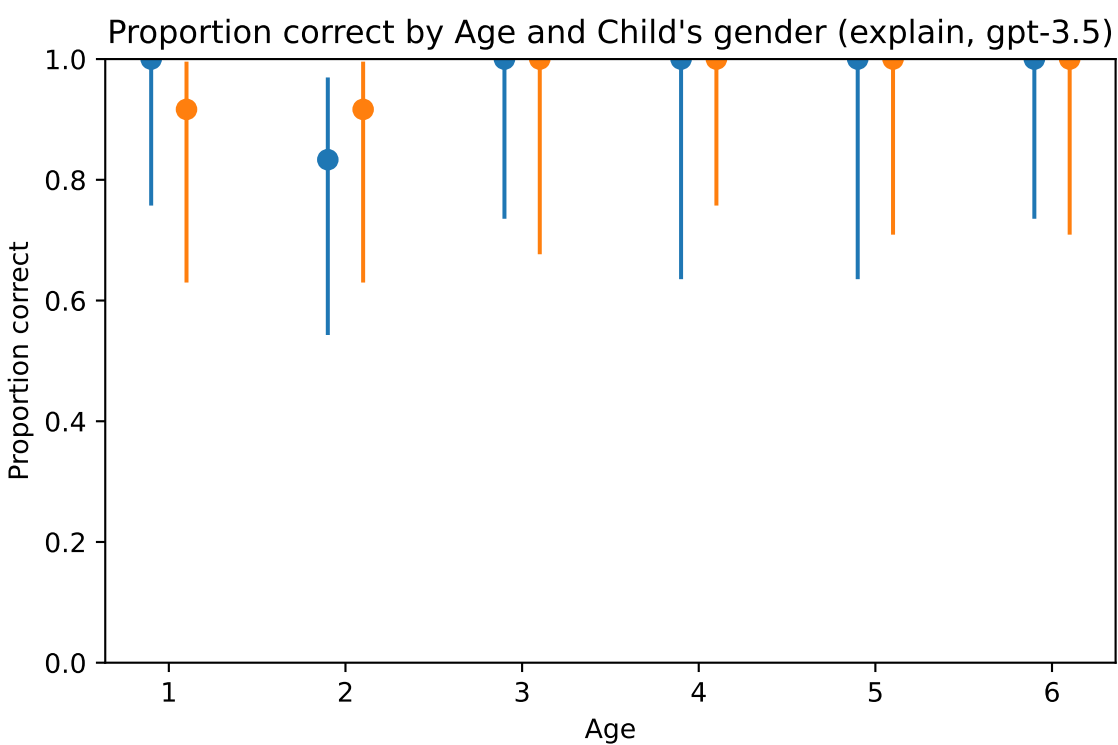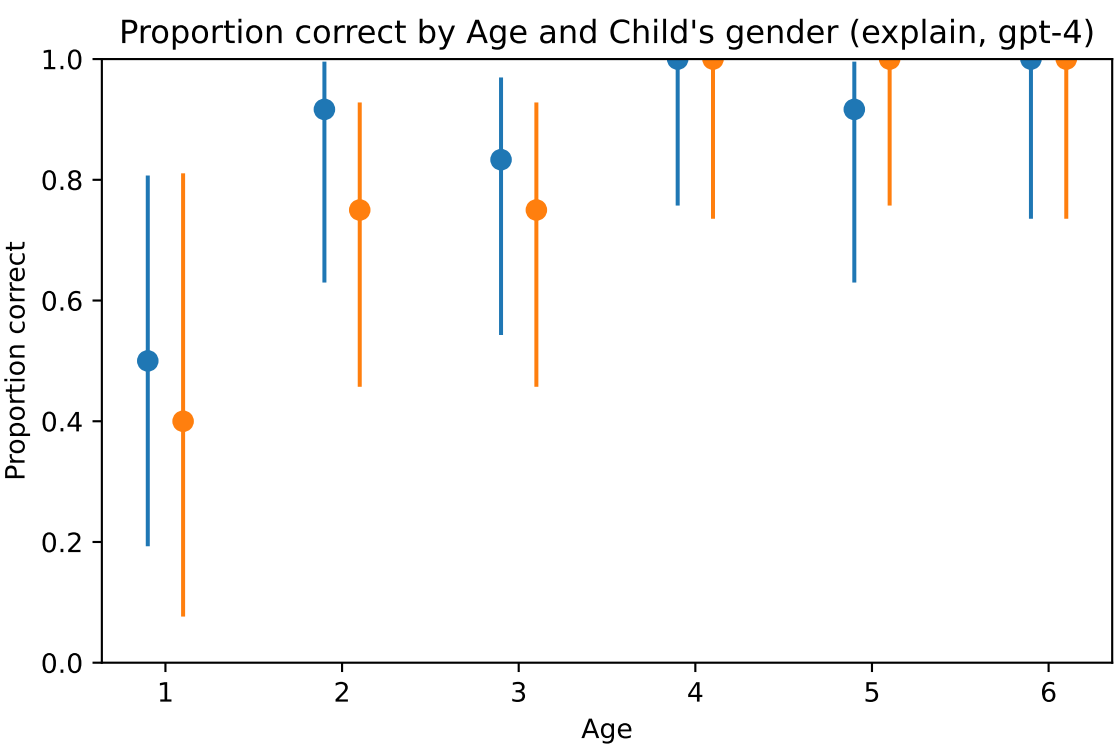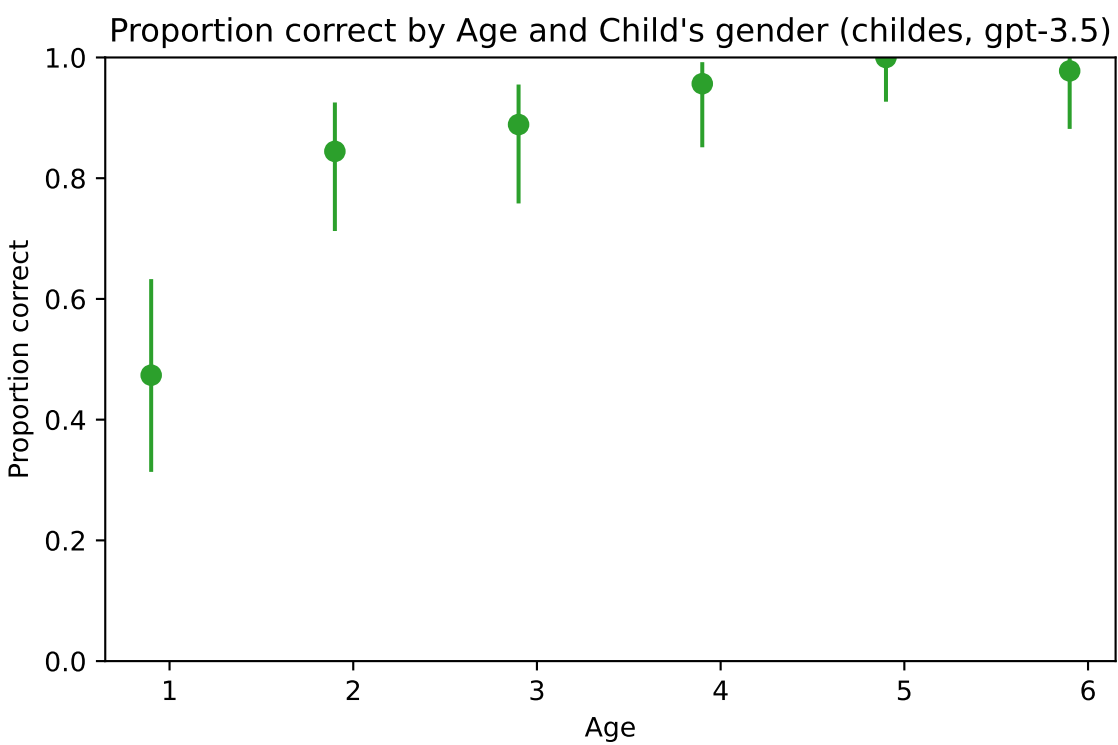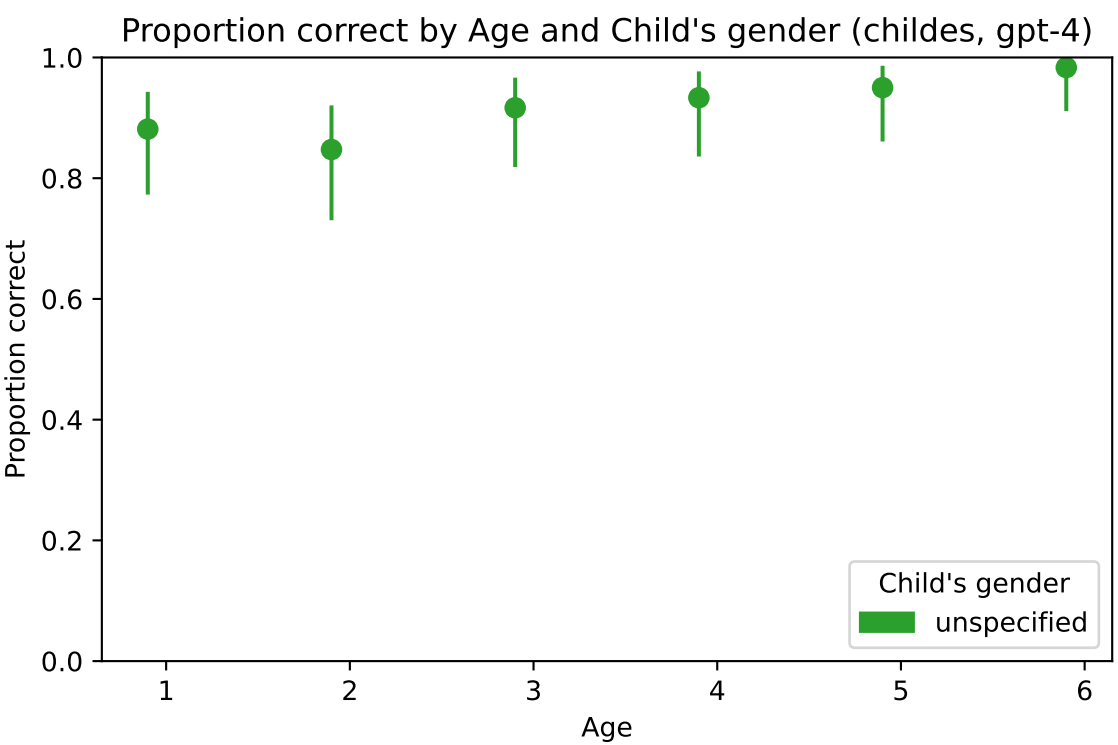

Supplement: S3 File — (ZIP) [file pone.0298522.s003.zip › S3 Figures/Proportion correct_Child's gender_relevant only.pdf]

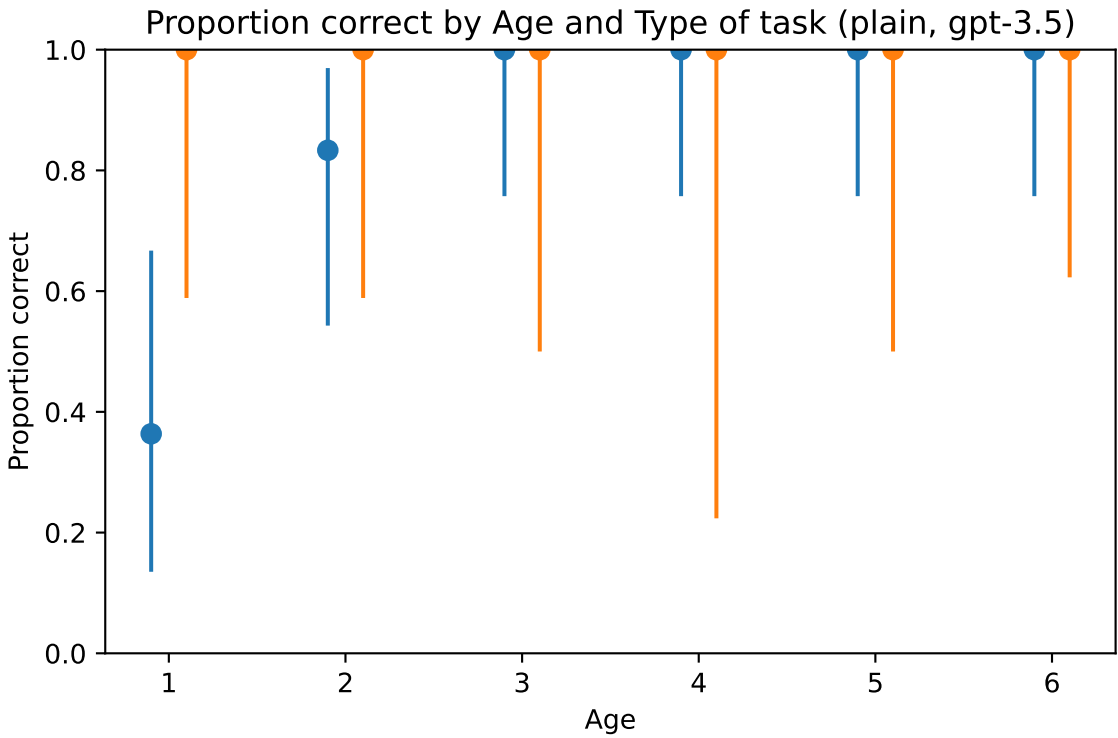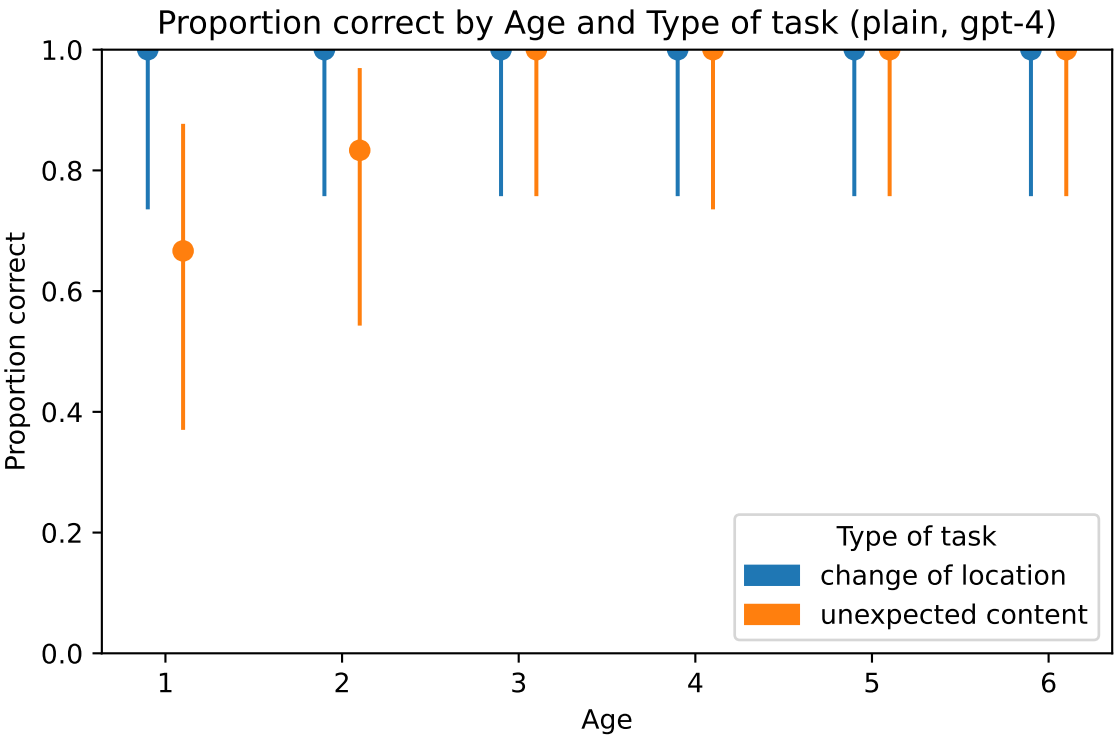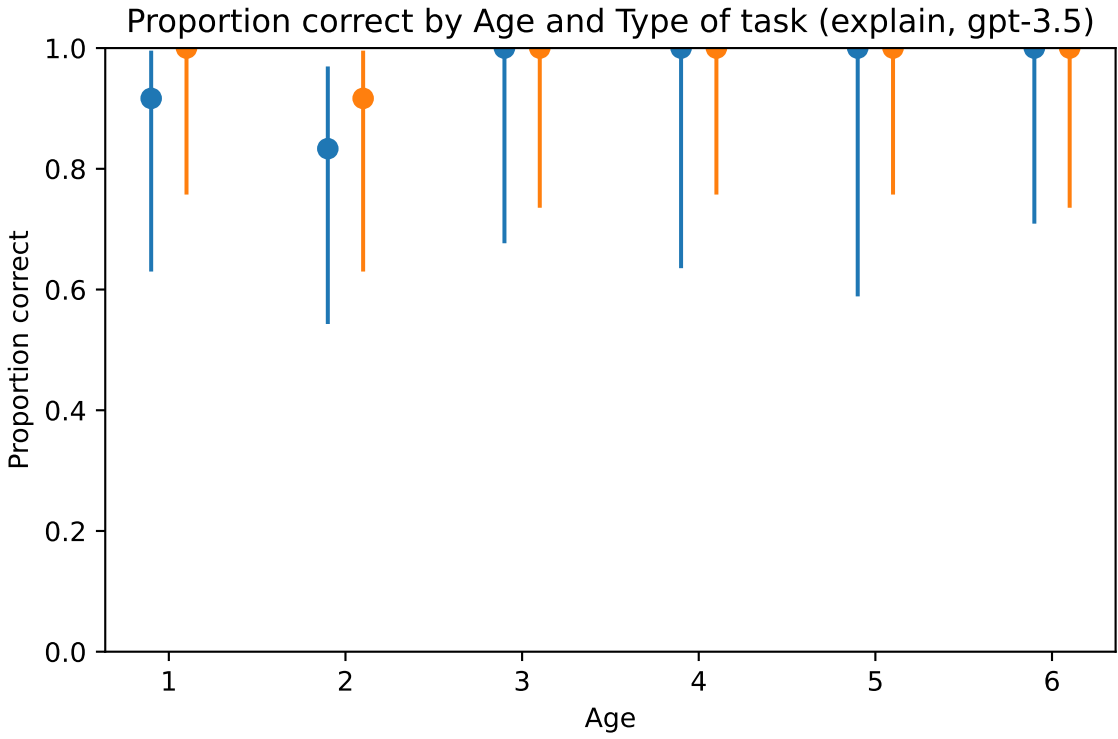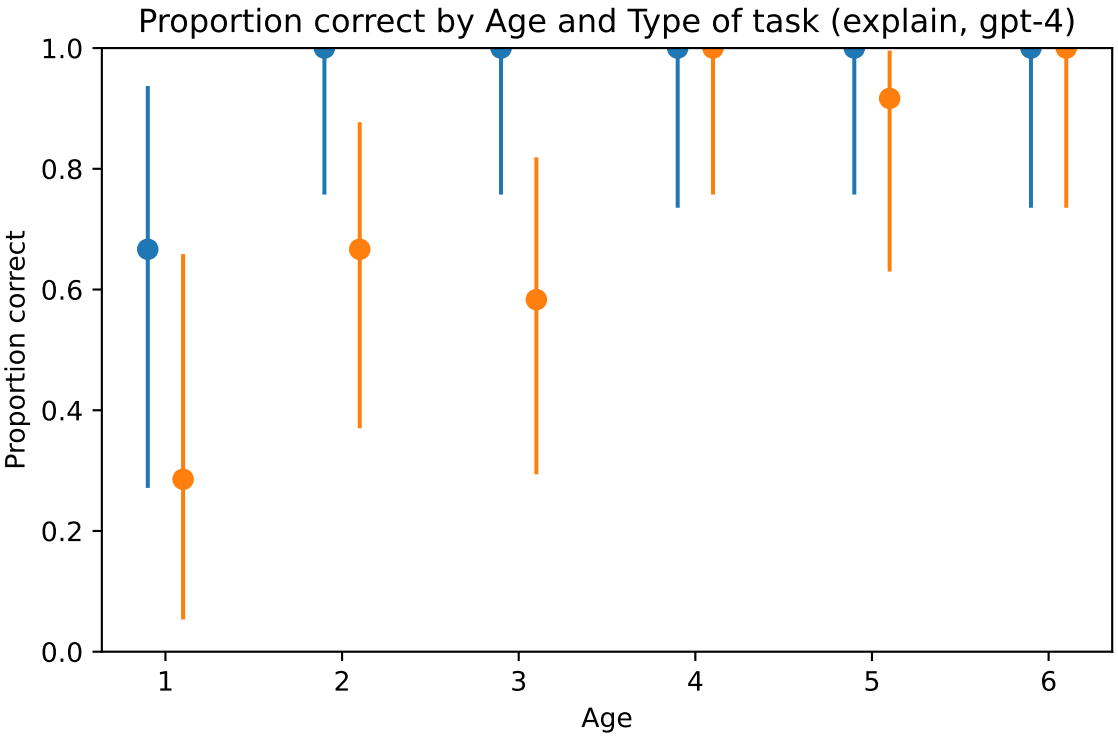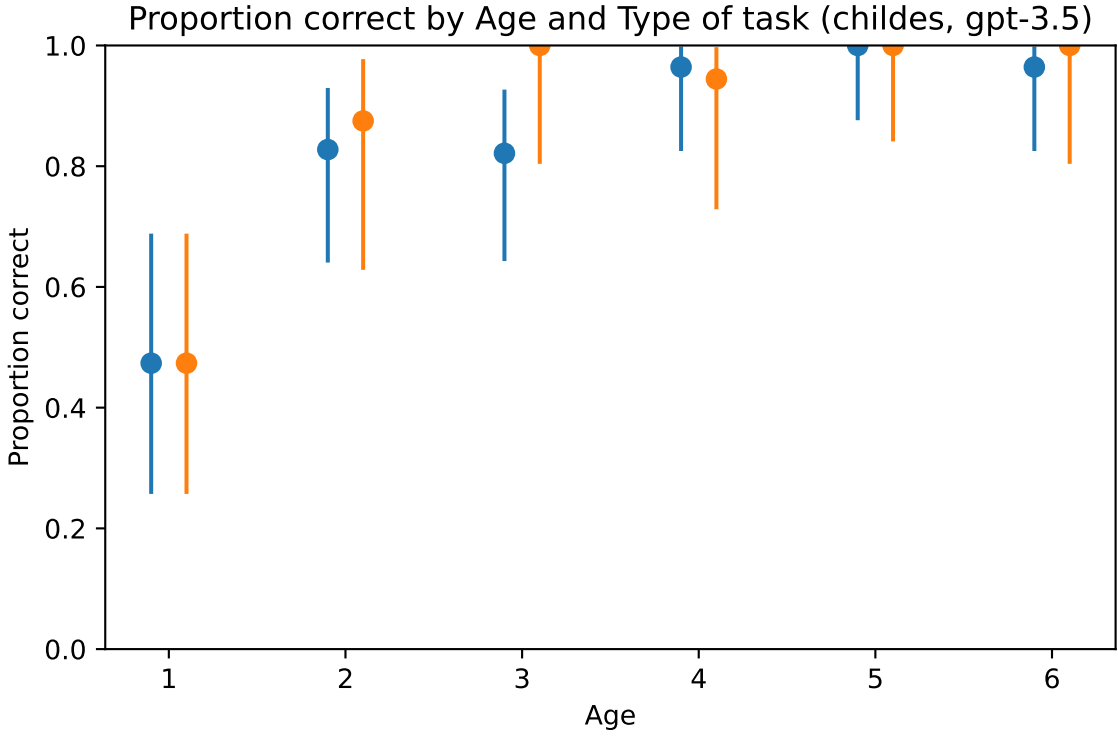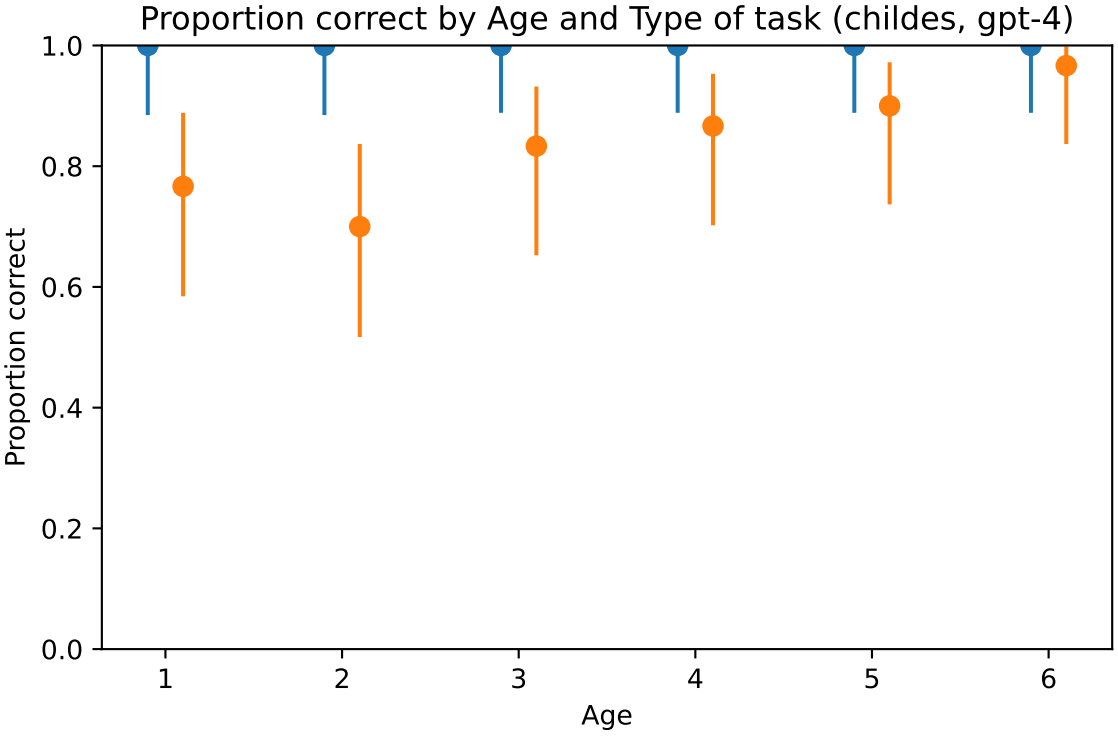

Supplement: S3 File — (ZIP) [file pone.0298522.s003.zip › S3 Figures/Proportion correct_Type of task_relevant only.pdf]

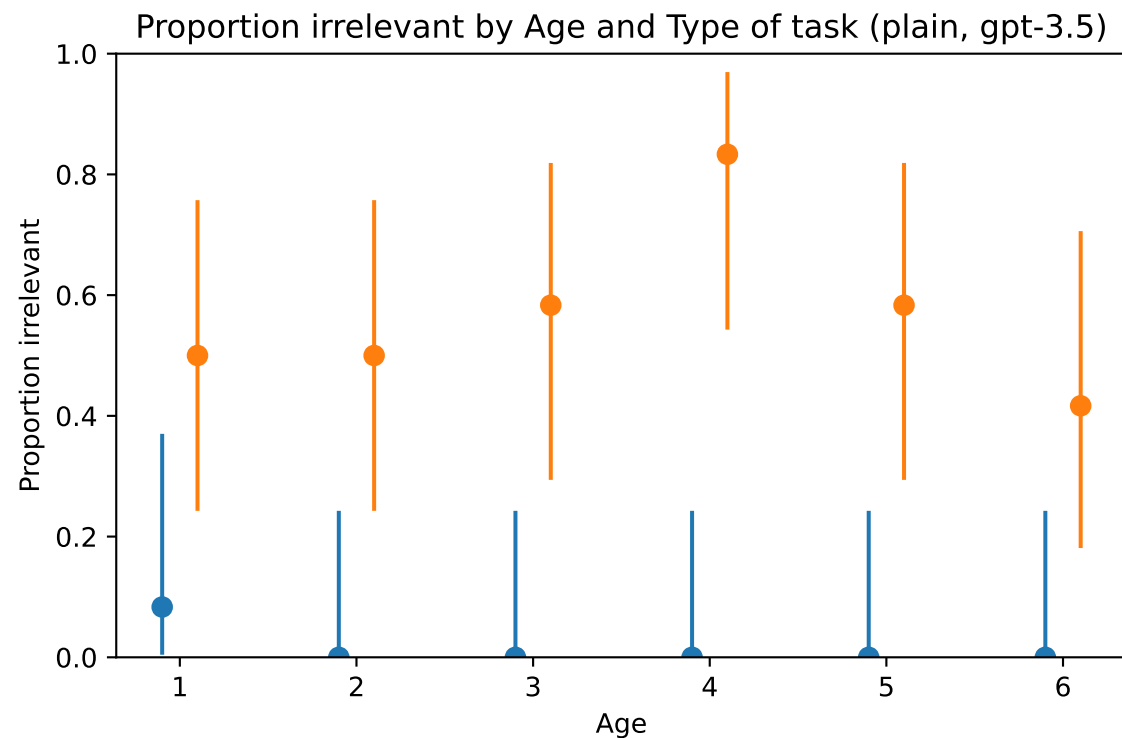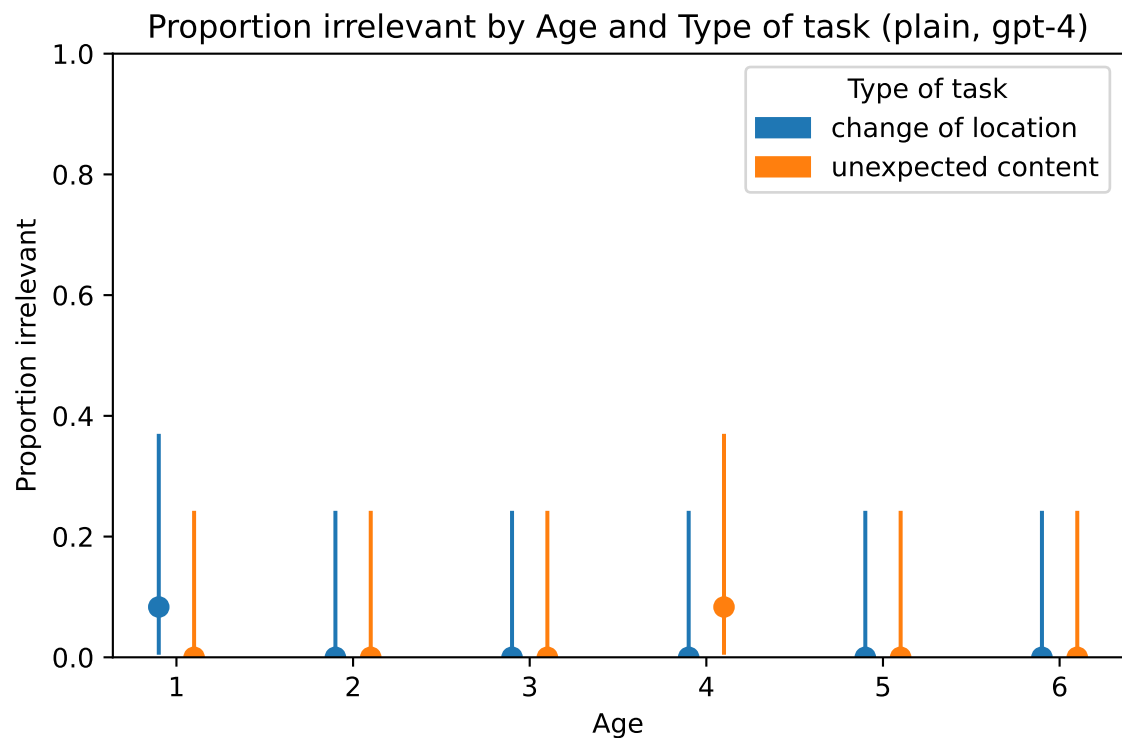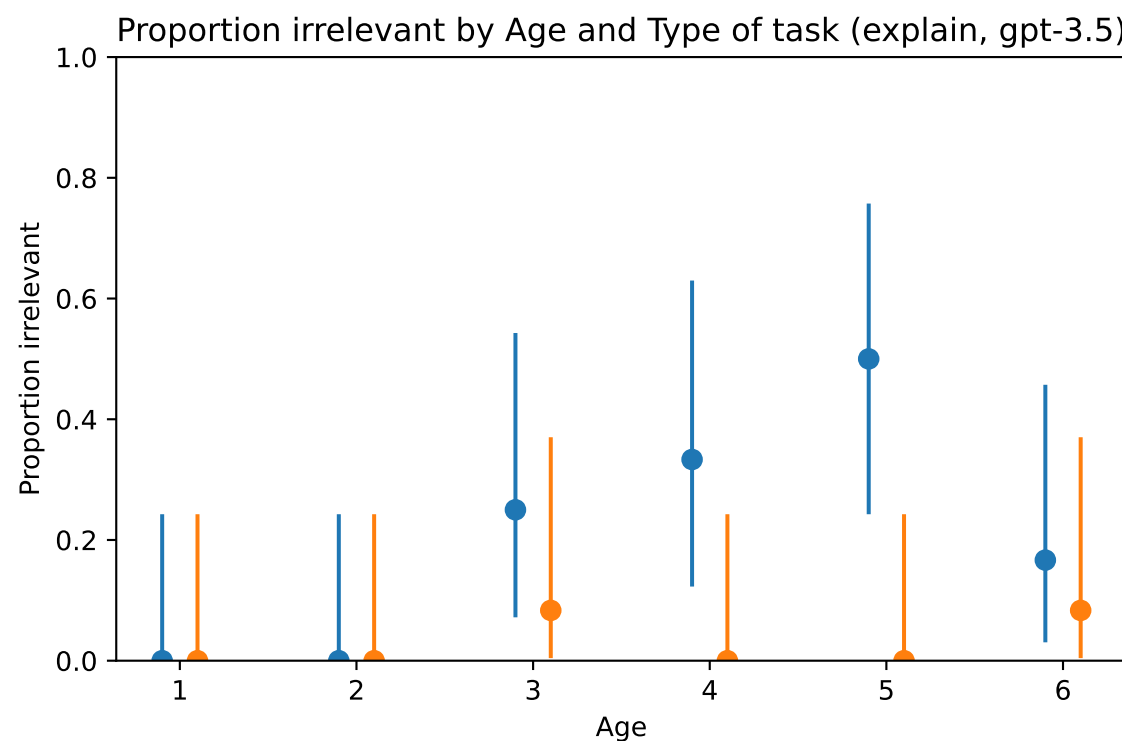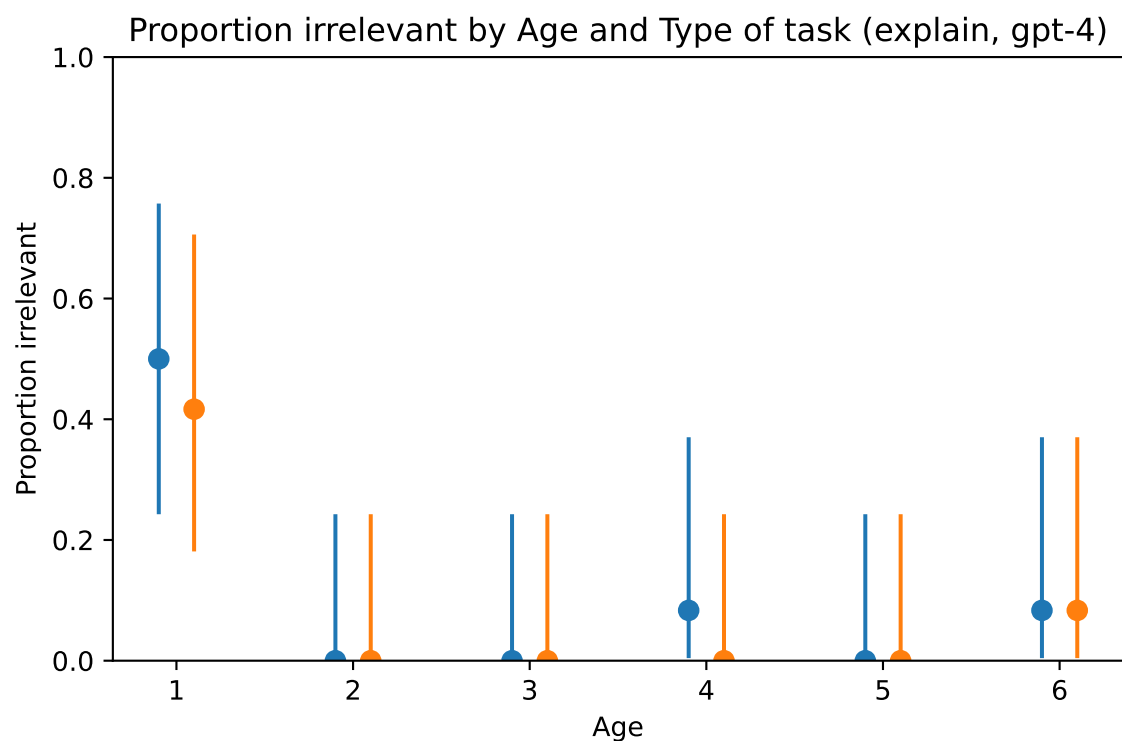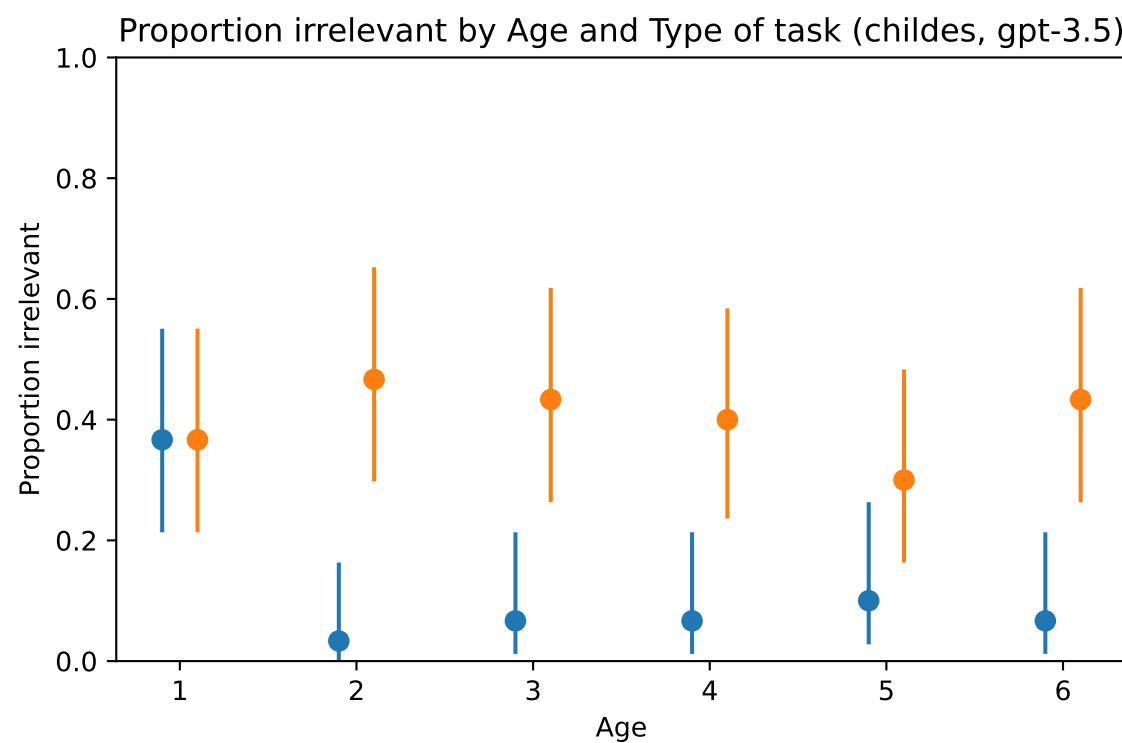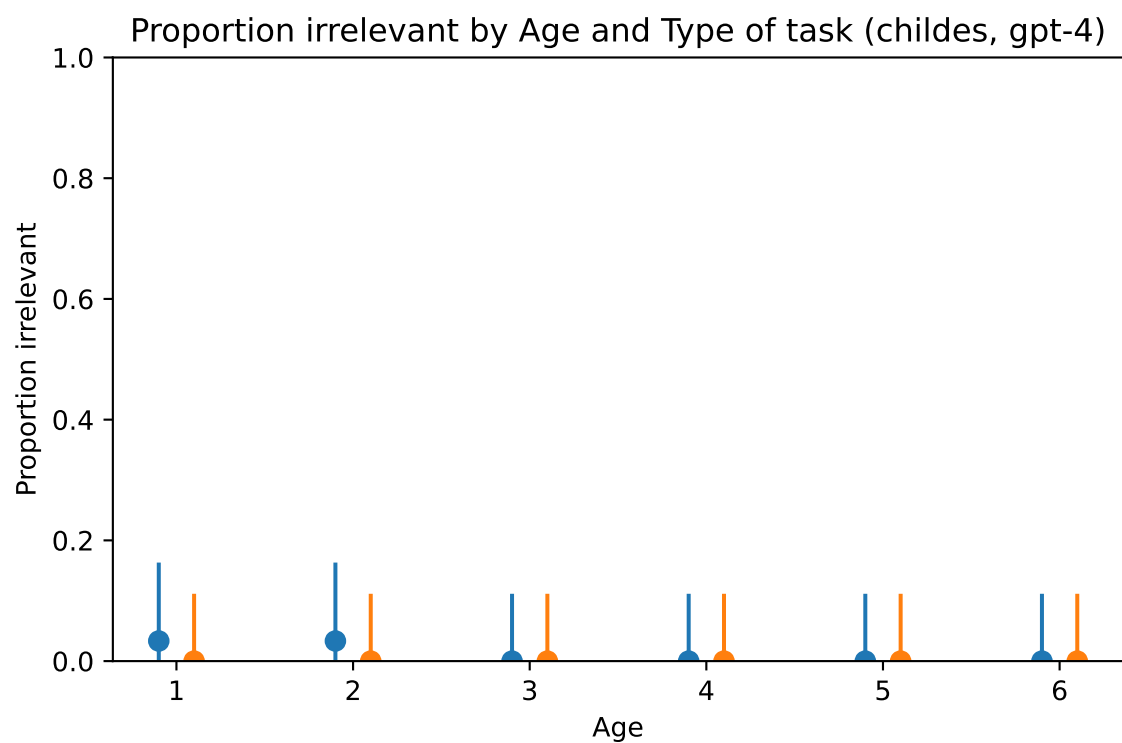

Supplement: S3 File — (ZIP) [file pone.0298522.s003.zip › S3 Figures/Proportion irrelevant_Type of task.pdf]
